# NPM promotes hepatotoxin-induced fibrosis by inhibiting ROS-induced apoptosis of hepatic stellate cells and upregulating lncMIAT-induced TGF-β2

**DOI:** 10.1038/s41419-023-06043-0

**Published:** 2023-08-30

**Authors:** Xue Ding, Xin-Le Zhu, Dong-Hui Xu, Shuang Li, Qiong Yang, Xian Feng, Yong-Gui Wei, Huan Li, Ling Yang, Yu-Jun Zhang, Xiao-Ling Deng, Kuan-Can Liu, Song-Lin Shi

**Affiliations:** 1 Cancer Research Center, School of Medicine, Xiamen University, Xiamen, China; 2 Department of Basic Medical Sciences, School of Medicine, Xiamen University, Xiamen, China; 3 Department of Hepatic Biliary Pancreatic Vascular Surgery, the First Affiliated Hospital of Xiamen University, School of Medicine, Xiamen University, Xiamen, China; 4 Xiang’an Hospital of Xiamen University, School of Medicine, Xiamen University, Xiamen, China

**Keywords:** Liver fibrosis, Translational research, Mechanisms of disease, Apoptosis

## Abstract

Liver fibrosis is caused by a variety of chronic liver injuries and has caused significant morbidity and mortality in the world with increasing tendency. Elucidation of the molecular mechanism of liver fibrosis is the basis for intervention of this pathological process and drug development. Nucleophosmin (NPM) is a widely expressed nucleolar phosphorylated protein, which is particularly important for cell proliferation, differentiation and survival. The biological role of NPM in liver fibrosis remains unknown. Here we show that NPM promotes liver fibrosis through multiple pathways. Our study found that NPM was up-regulated in cirrhosis tissues and activated in hepatic stellate cells (HSCs). NPM inhibition reduced liver fibrosis markers expression in HSCs and inhibited the HSCs proliferation and migration. In mice model, NPM knockdown in HSCs or application of specific NPM inhibitor can remarkably attenuate hepatic fibrosis. Mechanistic analysis showed that NPM promotes hepatic fibrosis by inhibiting HSCs apoptosis through Akt/ROS pathway and by upregulating TGF-β2 through Akt-induced lncMIAT. LncMIAT up-regulated TGF-β2 mRNA by competitively sponging miR-16-5p. In response to liver injury, hepatocytes, Kupffer cells and HSCs up-regulated NPM to increase TGF-β2 secretion to activate HSCs in a paracrine or autocrine manner, leading to increased liver fibrosis. Our study demonstrated that NPM regulated hepatotoxin-induced fibrosis through Akt/ROS-induced apoptosis of HSCs and via the Akt/lncMIAT-up-regulated TGF-β2. Inhibition of NPM or application of NPM inhibitor CIGB300 remarkably attenuated liver fibrosis. NPM serves a potential new drug target for liver fibrosis.

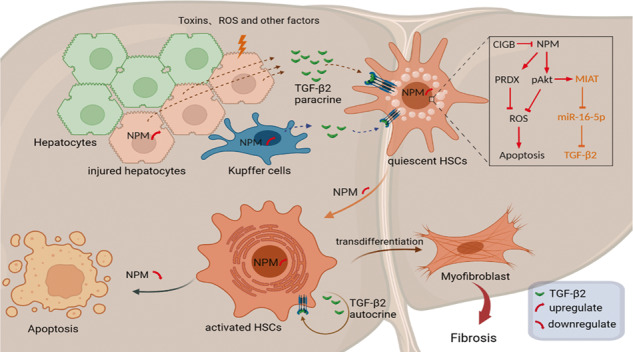

## Introduction

Hepatic fibrosis commonly results from multiple liver injuries and has become one of the leading causes of liver-related diseases and deaths worldwide [1]. Effective treatment for liver fibrosis is urgently needed. However, the mechanism of fibrosis has not been fully determined, thus limiting the development of anti-fibrosis drugs. So far, no anti-fibrosis drugs have been approved.

Liver fibrosis involves a variety of cells such as hepatic stellate cells (HSCs), hepatocytes, macrophages, endothelial cells and cholangiocytes. HSCs are the main effectors of fibrosis [2, 3]. HSCs are located in the interstitial space between sinus endothelial and hepatic epithelial cells, accounting for 5%–8% of liver tissue cells. HSCs are inactive in a normal healthy liver [4]. In response to liver injury, quiescent HSCs are activated by inflammatory factors or persistent oxidative stress from disordered hepatic microenvironment [5, 6]. Activated HSCs exhibit proliferation and migration characteristics, and are transformed into myofibroblast-like cells [7], secreting large amounts of collagen and extracellular matrix and causing hepatic fibrosis. HSCs produce almost 90% of the extracellular matrix and are major contributors to collagen deposition [8, 9].

The factors that activate HSCs include platelet-derived growth factor, transforming growth factor (TGF-β), tumor necrosis factor, and interleukin (IL) [10]. Among these factors, TGF-β1 is a powerful fibrogenic factor [11], which is mainly synthesized by HSCs/myofibroblasts, Kupffer cells, hepatic sinus endothelial cells, and hepatocytes [12]. The expression and activity of the three TGF-β isoforms are different in various cells of liver [13, 14], and the data of β2 and β3 isoforms in fibrotic diseases are still limited. TGF-β2 and TGF-β3 can be activated by an integrin-independent mechanism with a lower threshold than TGF-β1, and the inhibition of TGF-β2 and/or TGF-β3 alleviates fibrotic disease [15, 16]. However, little is known about the mechanisms that regulate TGF-β2 in fibrotic disease.

Nucleophosmin (NPM), a nucleolus phosphorylated protein, is widely expressed in proliferating cells, and is involved in a variety of cell biological processes [17–19]. NPM promotes proliferation, differentiation, and inhibits programmed cell death [20–22]. Most studies conducted so far have investigated NPM deregulation in cancers, and the high expression of NPM is associated with tumor progression [23, 24]. Whereas, the relevance between NPM and liver fibrosis has not been reported.

In the present study, we demonstrated that NPM promotes liver fibrogenesis by inhibiting the Akt/ROS-induced HSCs apoptosis and upregulating Akt/MIAT-induced TGF-β2 in HSCs, hepatocytes, and Kupffer cells. Moreover, NPM inhibitor hinders the progression of liver fibrosis in mice. Therefore, our results further shed light on the molecular mechanism of liver fibrosis and provide a potential new drug target for liver fibrosis.

## Materials and methods

### Statement

All research was conducted in accordance with both the Declarations of Helsinki and Istanbul, and all protocols were approved by the Ethics Committee on the Care and Use of Laboratory Animals of Xiamen University. All experiments and animal care were conducted based on the guidelines outlined in the National Institutes of Health Guide for the Care and Use of Laboratory Animals.

### Animal model of liver fibrosis

Five-week-old C57BL/6 mice (male, body weight: 18–20 g) were obtained from the Experimental Animal Center of Xiamen University. For the TGF-β2-treated mouse model, in addition to C57BL/6 mice, the experiment was repeated using Balb/c mouse, which contains more HSCs and may show more significant results. Liver fibrosis was induced via intraperitoneal injection of carbon tetrachloride (CCl_4_, 1 mL/kg body weight, diluted in corn oil, every 3 days) for 8 weeks. VA-coupled liposomes carrying siRNAs (VA-lip-siRNAs) were injected intravenously on the fifth week after the administration of CCl_4_. The siRNAs that were chemically modified with 2′-OMe and 5′-Chol consist of an equal mixture of two siRNAs that target NPM mRNA. A small amount of siRNAs used to show traces was chemically modified with 5′-FAM. Approximately 0.7 mg/kg body weight of siRNAs was injected every 3 days. CIGB300 [25] (10 mg/kg body weight, diluted in PBS, twice a week) was injected intravenously on the fifth week. The same volume of saline and corn oil was administered to the mice in both the normal and fibrosis control group. The mice in each group were sacrificed after 8 weeks.

The VA-lip-siRNAs that target HSCs were prepared as previously described [26] with some modifications. In the process, the freeze-dried empty liposomes (LipotrustTM EX Oligo, Lot No. HH00301) containing cationic lipid were prepared at a concentration of 1 mM by adding double-distilled water. To prepare VA-coupled liposomes that targets hepatic stellate cells, we mixed 200 nmol vitamin A (retinol, Sigma) dissolved in DMSO with 100 µL of liposome suspensions (100 nmol as cationic lipid) by vortex mixing in a 1.5 mL tube at 25 °C. Approximately 15 µL of siRNA mixture (114.84 µg siRNAs in DDW) was added to the retinol-coupled liposome solution with stirring at 25 °C. The ratio of siRNA-to cationic lipid was 1:11.5 (mol/mol), and the siRNA-to-liposome ratio (wt/wt) was 1:1. Any free retinol or siRNA was removed using an ultrafiltration centrifuge tube (Amicon Ultra concentrator 30,000 MWCO, Millipore) by centrifugation at 1500 × *g* at 25 °C. The material trapped in the filter was reconstituted with PBS to achieve the desired dose for in vivo use.

For in vivo administration, MK2206 was dissolved in a solution containing 10% DMSO,40% PEG300,5% Tween-80 and 45% saline and administered orally to mice at a dose of 240 mg/kg. The miR-16-5p mimics and NC mimics were prepared into VA-lip-RNAs complexes by the same method as the VA-lip-siNPM complex described above, and then injected into mice through the tail vein at a dose of 0.7 mg/kg, respectively.

### Histopathological analysis of liver tissues

The human liver disease spectrum tissue arrays (Lot No. LV20812a and LV805c) were obtained from US Biomax, Inc. The mouse liver tissues fixed with paraformaldehyde were dehydrated and paraffin-embedded. The liver tissues of different mice were arranged in the same paraffin blocks and sectioned into tissue arrays. These tissue arrays were stained with conventional Hematoxylin-Eosin or Sirius Red and photographed under a microscope. For immunohistochemical (IHC) analysis, after routine dewaxing and antigen repair, the tissue array was incubated with 3% hydrogen peroxide to inactivate endogenous peroxidase. After blocking with 5% BSA in PBS and incubation with antibodies at 4 °C overnight, the sheep anti-rabbit IgG-HRP (MXB Biotech, Fujian) was added the next day, and the samples were incubated for 30 min at 37 °C. Diaminobenzidine was used for color development. After the color reaction, hematoxylin was used for nuclear dyeing, gradient alcohol was used for dehydration, and neutral resin was used for sheet sealing.

For fluorescent-multiplex-IHC (mIHC) analysis, after incubation with HRP-IgG, the fluorophore-conjugated TSA®Plus amplification reagent was added, and the mixture was incubated for 10 min. The mIHC protocols of CST Company were used as basis for the specific procedures. The section was sealed with ProLong Gold antifade reagent before observation via confocal microscopy.

### Primary cell isolation

Primary cells were isolated from liver tissue of Balb/c mice aged 6–8 months by using conventional method. In the process, the mice were anesthetized with 10% chloral hydrate and soaked in 70% alcohol for 5 s, and the abdomen was opened after fixing the limbs. EGTA solution, Collagenase P (Roche), and Pronase E (Roche) solutions were injected into the liver through portal vein successively. After perfusion, the liver tissue was shredded and prepared into cell suspension, and the sample was digested by enzyme solution (including Collagenase P, Pronase E and DNase I) in a water bath at 37 °C for 15 min. After digestion, cell suspension was passed through 70-µm cell filter and centrifuged (250 g, 10 min) to obtain the cell precipitate.

For primary HSC isolation, the suspended cells were centrifuged (900 g, 15 min, 4 °C) at a density gradient with 15% and 35% Percoll solution. HSC layer was collected and inoculated in cell culture dish with D10 medium. After 2 h, the medium containing un-adherent cells was eliminated, and the remaining adherent cells were cultured with D10 medium. Cell purity was identified using the α-SMA immunofluorescence method.

For primary Kupffer cell isolation, the suspended cells were centrifuged (500 g, 15 min, 4 °C) at a density gradient with 25% and 50% Percoll solution. The Kupffer cell layer was collected and inoculated in cell culture dish. After 2 h, the medium containing un-adherent cells was eliminated. Cell purity was identified using the F4/80 immunofluorescence method.

### Rapid isolation of primary HSC in liver tissue and labeling of ROS and apoptosis probe

The liver tissue of a mouse was cut into tissue blocks of about 1 mm³ with a surgical blade. The tissue blocks were washed with 30 mL of pre-cooled PBS and centrifuged at 50 g for 1 min to remove the red blood cells in the supernatant. The tissue blocks were enzymolized with 15 mL digestive solution at 37 °C for 30 min, shaking from time to time. Enzymatic solution is prepared with DMEM, containing type IV collagenase (1 mg/mL), Streptomyces protease (1 mg/mL), DNase I (20 µg/mL) and 2% FBS. The single-cell suspension after enzymatic hydrolysis was put through a cell filter with a pore size of 70 µm into a 50 mL centrifuge tube, and pre-cooled PBS solution (containing 0.5% EGTA) was added to 40 mL to terminate enzymatic digestion. The cell suspension was centrifuged at 4 °C, 800 g for 5 min, and the supernatant was aspirated. 10 mL of pre-cooled ACK lysis buffer was added to the cell precipitate for suspension, and 40 mL of pre-cooled PBS was added two minutes later. After centrifugation, the supernatant was aspirated to remove the red blood cells.

1 mL PBS was added to the cell precipitate and fully suspended into a single-cell suspension (about 3 mL), which was evenly divided into two parts. Cell suspension I was added with ROS probe DCFH-DA to a final concentration of 5 µM and incubated in darkness at 37 °C for 30 min. While ROS labeling was performed on cell suspension I, hepatic stellate cells were isolated and labeled on cell suspension II. 1.5 mL of cell suspension II was centrifuged at 800 g for 2 min and the supernatant was aspirated. 12% Nycondens gradient separation solution (density of about 1.055-1.080 g/mL) prepared by GBBS was added to the cell precipitation to a volume of 13 mL, and fully suspended into a single-cell solution. About 2 mL D-Hanks (containing 2% FBS) was carefully added to the upper liquid surface along the tube wall. Centrifuge with a horizontal rotor at 1350 g for 10 min. Cells in the D-Hanks solution and at the interface of the two solutions were collected, washed with 5 times the volume of D-Hanks (containing 2% FBS), centrifuged at 600 g for 5 min, and the supernatant was aspirated. The above precipitated cells separated from cell suspension II are HSCs, which are further labeled with Annexin V-FITC Apoptosis Detection Kit (Beyotime, Lot No.C1062L). While labeling apoptotic cells, HSCs labeled with ROS probes can be separated from cell suspension I using the method described above. The whole process takes about 110 min, which preserves the activity of the cells and the in vivo physiological state to the greatest extent. The labeled and isolated HSCs were detected by flow cytometry for ROS and apoptosis respectively.

### Cell culture and drug treatment

The human HSC line LX-2 was obtained from BeNa Culture Collection of China and identified by Genetic Testing Biotechnology Corporation (Suzhou, China) by using short tandem repeat markers. The rat HSC line CSFC-2G was donated by Dr. Honghua Zheng from Xiamen University. The HepG2 and SK-HEP-1 human liver cancer cell lines and human embryonic kidney 293 T cell line were obtained from the Cell Bank of Shanghai Institutes for Biological Sciences of China. These cell lines were cultured in DMEM (Gibco, Cat#12800-017) containing 10% fetal bovine serum (PAN, Cat#ST30-3302). The THP-1 cell line was cultured in RPMI-1640 medium (BasalMedia, Cat#L210K). The drugs involved in this study include TGF-β1 (PeproTech, Cat#100-21-10), TGF-β2 (Novoprotein, Cat#CJ79-50), NPM inhibitor CIGB300 (Royo Biotech, Shanghai), Akt phosphorylation inhibitor MK2206 and activator SC79 (Selleck, Cat# S1078, Cat#S7863), and ROS inhibitor N-acetyl-cysteine (NAC) (Beyotime, Cat#ST1546). TGF-β1 (10 ng/mL), TGF-β2 (10 ng/mL), CIGB300 (4 and 8 µM), MK2206 (4 and 8 µM), SC79 (4, 8, and 12 µM), and NAC (1 and 3 µM) were used for cell treatment.

### NPM knockout cell lines

The pCW-Cas9 plasmid and pLX-sgRNA (target sequence: GCAAGAATCCTTCAAGAAAC) were co-transfected into cells, and the expression of Cas9 was induced using 2 µg/mL doxycycline after 24 h of culture. Puromycin at 1.5 µg/mL and Blasticidin S at 0.85 µg/mL were added after 24 h of culture. Resistant clonal communities were observed after culture for 1–2 weeks. At this time, monoclonal cells were selected and sub-cultured for two generations, and a sufficient number of cells was collected for verification via Western blot and PCR.

### NPM knockdown and MIAT expression interference

The sequences of siRNA, shRNA, sgRNA, and negative control are listed in Supplementary Tables S1 and S2 in the supplementary material. The shRNA vector was constructed using pLKO.1 plasmid and packaged as lentivirus to infect cells and knockdown gene expression. The sgRNA vector for MIAT transcription activation was constructed using lentiSAM v2 (Puro) plasmid, which was provided by Adam Karpf (Addgene plasmid #92062).

### Immunoblotting

The protein was transferred to PVDF membrane (Millipore), and the membranes were incubated with the primary antibody, and then with horseradish peroxidase-conjugated secondary antibody against IgG. Membranes were exposed with ECL reagent (Thermo Fisher) and analyzed by Chemiluminescence imaging system Azure 300 (Azure Biosystems, USA). The antibodies used in this study are listed in Supplementary Table S3, and the original images of Western blot are provided in Supplementary Materials.

### Real-time quantitative PCR

Total RNA was extracted using TRIzol and was reverse-transcribed into cDNA by using PrimeScript RT reagent kit with gDNA Eraser (TaKaRa, Cat#:RR047A). Subsequently, cDNA was used as template for PCR amplification on the ABI7500 instrument by using ChamQ SYBR qPCR Master Mix (Vazyme, Cat# Q331-03).

### MIAT ISH and RNA-Protein co-detection

The cells seeded on the slide or the fresh frozen tissue sections were subsequently fixed with paraformaldehyde, inactivated with hydrogen peroxide, antigenic repaired with citric acid buffer, and treated with protease IV. Hybridization was performed using the MIAT probe (ACD, Cat # 458501), and the sample was labeled with TSA®Plus fluorescent dye. For the dual labeling process of RNA in situ detection and immunohistochemistry staining, antibody staining was performed after RNA hybridization. Details are provided in the user manual of the RNAscope® Multiplex Fluorescent V2 (Cat #323100, Advanced Cell Diagnostics, Inc.).

### Cell proliferation assay

Logarithmically grown cells were seeded in 96-well plates with 4000 cells in each well and five replicates in each group. After the cells were completely adhered to the wall (6–10 h), the first time point was measured. Approximately 10 µL of CCK-8 solution (Beyotime, Shanghai) was added to each well. The cells were incubated at 37 °C with 5% CO_2_ for 4 h. The absorbance at 450 nm was determined using a microplate reader.

### ROS detection

CellROX Orange Reagent (Yeasen) was used to label intracellular hydrogen peroxide (H_2_O_2_), and dihydroethidium (Beyotime) was used to label intracellular superoxide anion (O_2_^-^). The fluorescent probe was diluted with serum-free medium to a final concentration of 5 mM, added into adherent cultured cells, and incubated at 37 °C for 30 min. The cells were washed thrice with PBS, and then re-suspended in PBS. The cell fluorescence was detected by flow cytometry with excitation wavelength of 561 nm and emission wavelength of 620 nm.

### Apoptosis detection

LX-2 cells were starved in serum-free medium for 12 h and stimulated by CIGB300 at different concentrations (2, 4, 6, 8, and 10 µM) or MK2206 (4 and 8 µM) for 6 h. CIGB300 was dissolved in PBS. MK2206 was dissolved in DMSO and diluted in PBS. The cells in the control group(s) were treated with PBS or diluted DMSO in PBS. Cells were harvested, and Annexin V-FITC and PI were added and incubated at room temperature in the dark for 15 min. Cell apoptosis was detected by flow cytometry.

### Statistical analysis

Statistical methods were not used to determine the sample size in advance. The mice were randomly divided into experimental groups. The injection was not blind. There are no exclusion criteria for animals. Graphpad Prism 7.0 (GraphPad, San Diego, USA) was used for all statistical analysis. Data were expressed as mean ± SEM. The variance was similar between the groups that were being statistically compared. The significance of differences between two groups were evaluated by standard t test when the variance is similar between the groups. Significance was defined at *P* value < 0.05. All experiments were performed in triplicate at least three times and representative data are shown.

## Results

### NPM is highly expressed in fibrotic liver tissue and activated HSCs

**Fig. 1:**
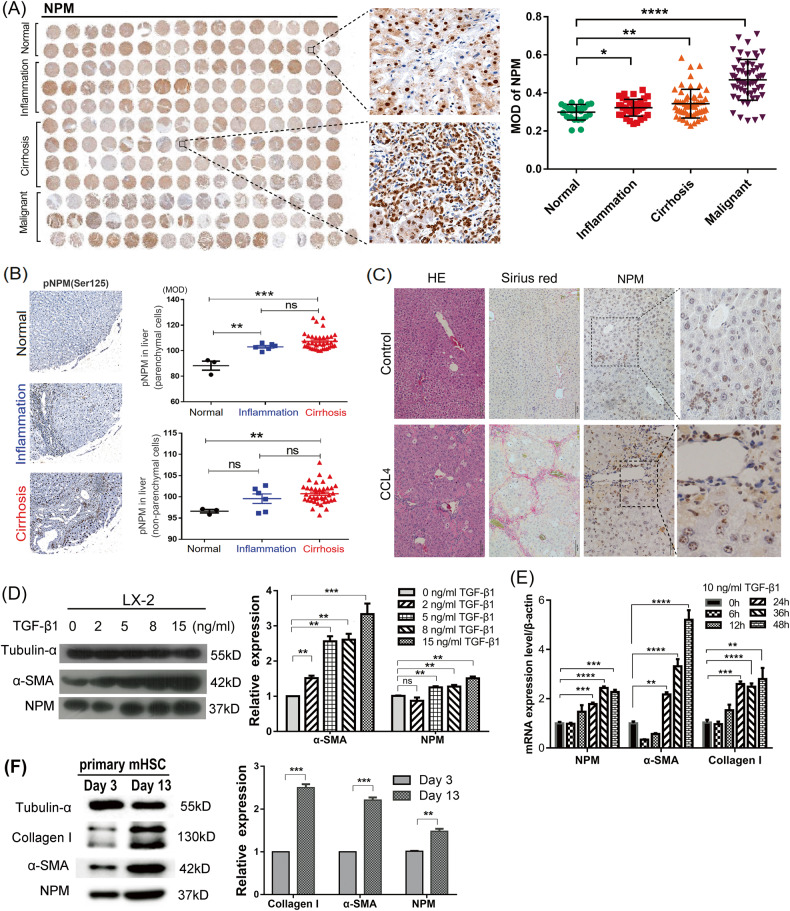
NPM is highly expressed in fibrotic liver tissue and activated hepatic stellate cells. **A** IHC staining results indicated a significant increase of NPM protein in the inflammatory and fibrotic liver and the liver cancer tissue of human liver disease spectrum tissue arrays (LV20812a), involving 32 cases of normal liver tissue, 48 cases of hepatitis, 72 cases of hepatic cirrhosis, and 48 cases of malignant tumor. **B** Quantitative analysis results of human liver tissue microarray (LV805c) confirmed that NPM (Ser125) phosphorylation was significantly increased in liver parenchymal and non-parenchymal cells in the cirrhosis stage. **C** A mouse model of liver fibrosis induced by CCl_4_ confirmed that NPM remarkably increased during cirrhosis. Hematoxylin-Eosin (HE) and Sirius red staining of liver tissues suggested significant fibrosis changes in liver tissues induced by CCl_4_ (*n* = 5). Immunohistochemistry staining showed that NPM remarkably increased in the liver tissues of CCl_4_-treated mice. **D** After starvation in serum-free medium for 24 h, TGF-β1 was used to stimulate LX-2 cells for 48 h, and Western blot showed increased protein expression of NPM and α-SMA. **E** Quantitative PCR showed that α-SMA and NPM mRNAs increased after TGF-β1 treatment. **F** The isolated primary mouse HSCs were activated, and the expression of NPM increased during culture. The data shown represent the result of three independent experiments. Expression data of WB or qPCR were relatively quantified according to the expression levels of β-actin or α-tubulin gene. Mean ± SEM; *p < 0.1, **p < 0.01, ***p < 0.001, ****p < 0.0001; *t* test.

Our previous study showed that NPM was significantly increased in cirrhotic tissues of rats, suggesting that NPM may be involved in liver fibrosis [27]. In the present study, the relevance between NPM and human liver fibrosis was determined in human liver disease tissue microarray. IHC staining showed that NPM is up-regulated in inflammatory and cirrhotic tissues (Fig. 1A), and the phosphorylation of NPM protein at Ser125 was significantly up-regulated in liver parenchymal cells or non-parenchymal cells (Fig. 1B) of cirrhosis tissue, suggesting that NPM is related to human liver fibrosis progression.

In mice with CCl_4_-induced liver fibrosis, HE and Sirius red staining showed significant fibrosis changes in liver (Fig. 1C). With fibrosis, IHC results showed that NPM in liver tissues remarkably increased. Therefore, NPM and mouse liver fibrosis are correlated, and NPM may be involved in the progression of liver fibrosis.

Since HSCs are the main effector cells of hepatic fibrosis, and their activation and collagen secretion are the central events of hepatic fibrosis, we first focused on HSCs and analyzed the relevance between NPM expression and HSC activation. The TGF-β1-induced activation of HSC LX-2 is a widely used hepatic fibrosis cell model. Western blot and quantitative PCR analysis showed that both fibrosis markers α-SMA and NPM increased in a dose-dependent manner after treatment with TGF-β1 (Fig. 1D, E). Considering that LX-2 is an activated HSC, we examined NPM changes in primary isolated mouse HSCs during activation. WB results showed that primary HSCs were activated gradually during culture, and NPM increased significantly with the increase in α-SMA and collagen I (Fig. 1F).

### Silencing or functional inhibition of NPM significantly impeded the expression of fibrosis markers and inhibited the proliferation and migration of HSCs

**Fig. 2:**
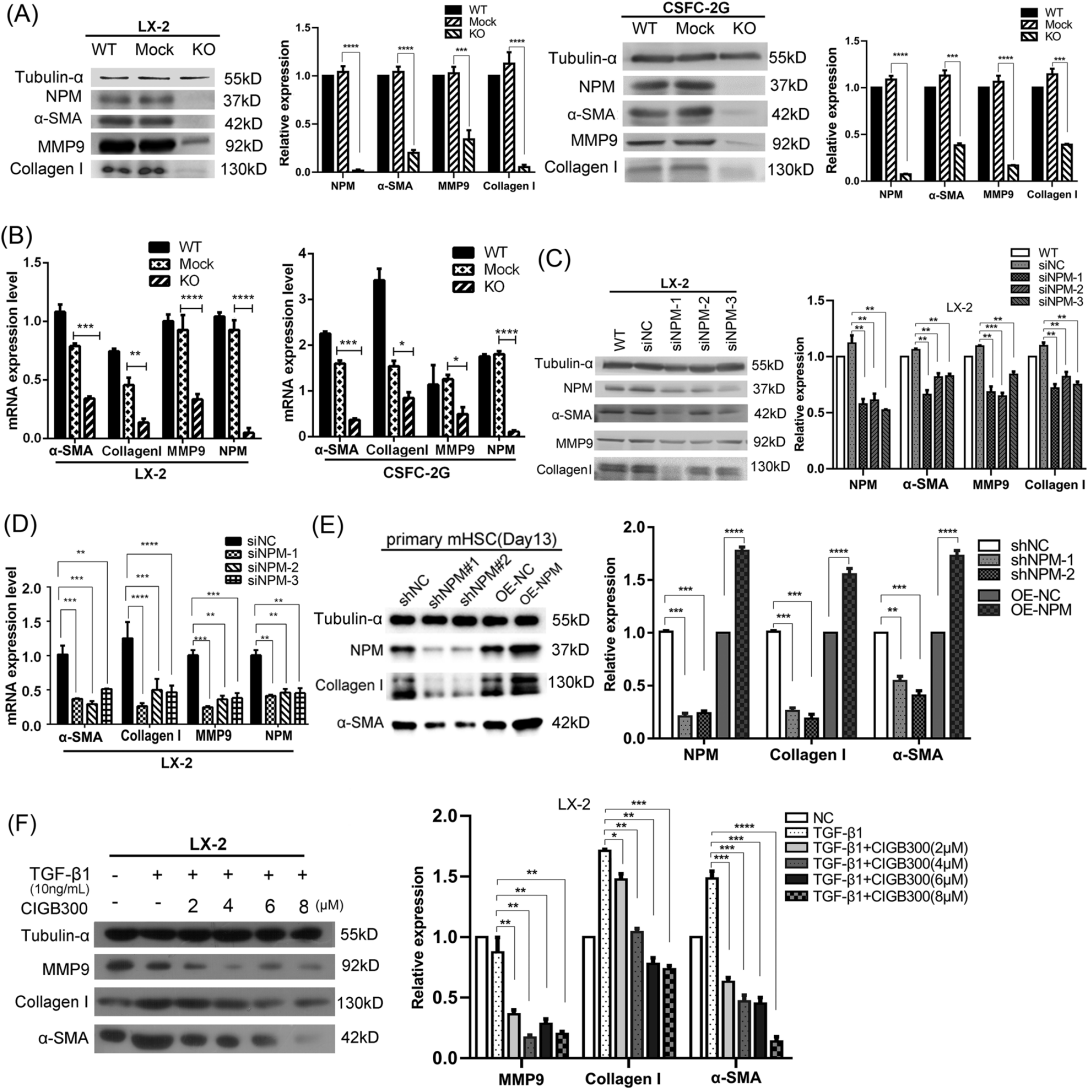
Silencing or functional inhibition of NPM in LX-2 cells significantly decreased the expression of liver fibrosis markers α-SMA, collagen I, and MMP9. **A** Western blot results showed that when NPM in CSFC-2G and LX-2 cells was knocked down by CRISPR-Cas9, the expression levels of α-SMA, collagen I, and MMP9 were sharply downregulated. **B** Quantitative PCR confirmed the decreased mRNA expression of α-SMA, collagen I, and MMP9 in LX-2 and CSFG-2G cells with NPM knocked out. **C** Western blot analysis results showed that NPM knockdown by siRNAs decreased fibrosis markers, including α-SMA, collagen I, and MMP9 in LX-2 cells. **D** Quantitative PCR further confirmed the NPM knockdown by siRNAs and the reduced α-SMA, collagen I, and MMP9. **E** NPM protein was knocked down or overexpressed in primary hepatic stellate cells cultured for 13 days, and markers of liver fibrosis were decreased or increased correspondingly. **F** CIGB300, a small molecule inhibitor of Ser125 phosphorylation of NPM protein, downregulated α-SMA, collagen I, and MMP9 in LX-2 cells. The data shown are representative of three independent experiments and relative quantification of data based on β-actin or α-tubulin gene expression. *n* ≥ 3; mean ± SEM; **p < 0.01, ***p < 0.001, ****p < 0.0001; *T* test.

To determine the role of NPM in the activation of HSCs, CRISPR-Cas9 and small interfering RNA (siRNA) techniques were used to silence the expression of NPM in human (LX-2) and rat HSCs (CSFC-2G), respectively. When the NPM in CSFC-2G and LX-2 cells was completely knocked out, the expression of fibrosis markers α-SMA, collagen I, and MMP9 was remarkably downregulated (Fig. 2A, B). NPM knockdown by siRNAs also decreased fibrosis markers to varying degrees (Fig. 2C, D). NPM protein was further knocked down or overexpressed in primary mouse HSCs cultured for 13 days. With the change of NPM, liver fibrosis markers also decreased or increased correspondingly, suggesting that NPM affects the level of liver fibrosis markers (Fig. 2E). Western blot results show that a small molecule CIGB300, which could bind to NPM protein and inhibit phosphorylation at Ser125 [25, 28], dose-dependently inhibited α-SMA, collagen I, and MMP9 in LX-2 cells (Fig. 2F). The α-SMA is a marker for the transdifferentiation of HSCs into myofibroblasts. Therefore, NPM can significantly affect the transdifferentiation of HSCs and the synthesis of collagen.

Proliferation and migration are important features of HSC activation. Accordingly, we examined the effect of NPM on the proliferation and migration of HSCs. The results of CCK-8 proliferation assay showed that NPM knockout in CSFC-2G cells (Supplementary SFig. 1A) or NPM knockdown in human LX-2 cells (SFig. 1B) significantly inhibited cell proliferation. Transwell and Scratch assays showed that NPM silencing significantly inhibited the migration of CSFC-2G (SFig. 1C) and LX-2 (SFig. 1D) cells.

### NPM knockdown in HSCs significantly inhibited hepatic fibrosis in mice

**Fig. 3:**
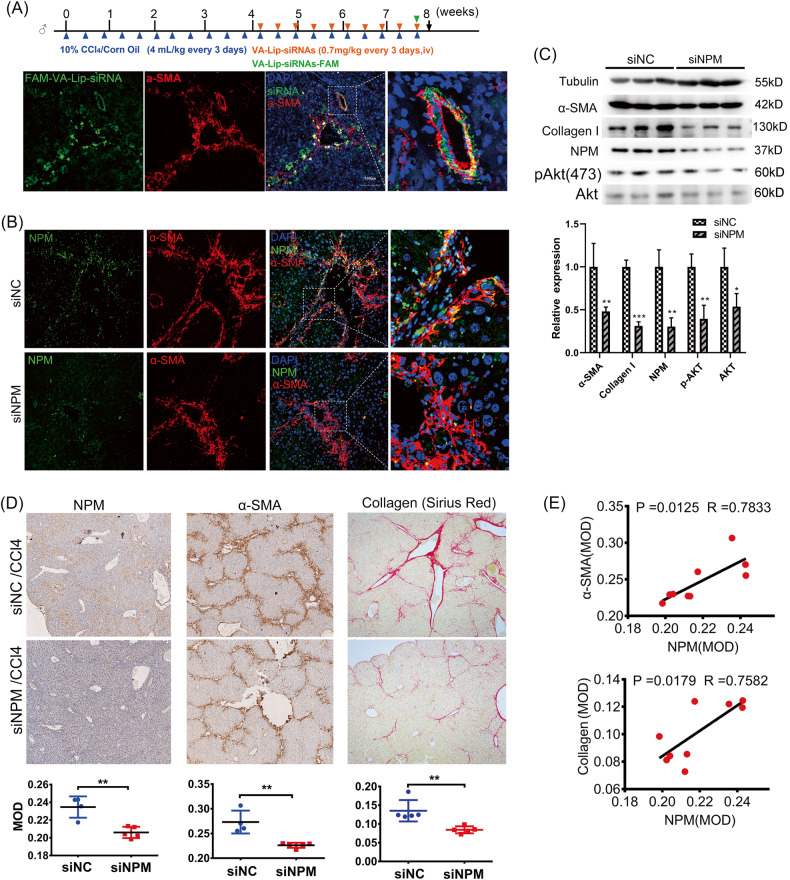
Targeted knockdown of NPM in mouse hepatic stellate cells (HSCs) significantly inhibited the progression of hepatic fibrosis in mouse models induced by carbon tetrachloride. **A** NPM expression of activated HSCs in mouse liver was knocked down by a mouse HSC-specific VA-lip-siRNA complex via tail vein injection. Representative fluorescent images of α-SMA (red) and VA-lip-siRNA-FAM (green) in liver specimens from carbon tetrachloride-treated cirrhotic mice indicated that the VA-lip-siRNA-FAM was specifically enriched in HSC. The mice (*n* = 5 /group) were injected with VA-lip-siNPM or VA-lip-siNC every 3 days (10 injections in total). The liver specimens were harvested 24 h after the last injection of VA-lip-siRNA-FAM. **B** Yellow co-location fluorescence of NPM and α-SMA showed that siNPM significantly downregulated NPM expression in HSCs. **C** Western blot analysis of liver tissues confirmed that NPM was significantly increased in liver. The expression levels of α-SMA, collagen I, and phosphorylated Akt were significantly decreased. **D** IHC staining and Sirius red staining confirmed that NPM level was significantly decreased by VA-lip-siNPM, and the expressions of markers of liver fibrosis, such as α-SMA and collagen (Sirius red staining), were significantly reduced. **E** Pearson correlation test showed that NPM was positively correlated with α-SMA and collagen I. Mean ± SEM; **p < 0.01; *T* test.

The retinol-coated liposomes can effectively target HSCs for siRNA delivery because of their remarkable capacity for vitamin A uptake. The enrichment percentages of siRNA carried by retinol-coated liposomes (VA-Lip-siRNA) in primary HSCs, macrophages, and hepatocytes were 32.96%, 1.65%, and 0.21%, respectively, showing strong specificity for HSCs [26]. To demonstrate that NPM in HSCs affects fibrosis, the VA-Lip-siNPM complex were injected into the tail vein of CCl_4_-induced hepatic fibrosis mice to knockdown NPM in HSCs (Fig. 3A). An equal mixture of two siRNAs targeting NPM was used to reduce potential off-target effects. The enrichment effect of VA- Lip-siRNA in HSCs was evaluated using FAM-labeled siRNA as tracer molecule. Confocal microscopy observation showed that FAM-labeled siRNA was mainly enriched in the portal region and had a significant co-localization with α-SMA, suggesting the specific targeting effect of VA-Lip-siRNAs on HSCs (Fig. 3A). Fluorescence analysis showed that NPM protein in HSCs was significantly downregulated by siNPM (Fig. 3B). Western blot analysis of mouse liver tissue showed that the expression of α-SMA, type I collagen, and phosphorylated Akt, were also downregulated with NPM knockdown (Fig. 3C). IHC staining and Sirius red staining of mouse liver tissue also confirmed the downregulation of α-SMA and type I collagen (Fig. 3D). The expression of NPM was positively correlated with α-SMA and collagen (Fig. 3E). Therefore, NPM knockdown in HSCs significantly inhibited CCl_4_-induced hepatic fibrosis in mice.

### CIGB300, an NPM inhibitor, significantly inhibited liver fibrosis in mice

NPM is a multifunctional protein, so can directly inhibit the function of NPM protein attenuate the fibrosis? To answer this question, CIGB300, a functional inhibitor of NPM protein with a transmembrane peptide structure, was dissolved in PBS and administered it via tail vein to the CCl4-induced liver fibrosis mice.

**Fig. 4:**
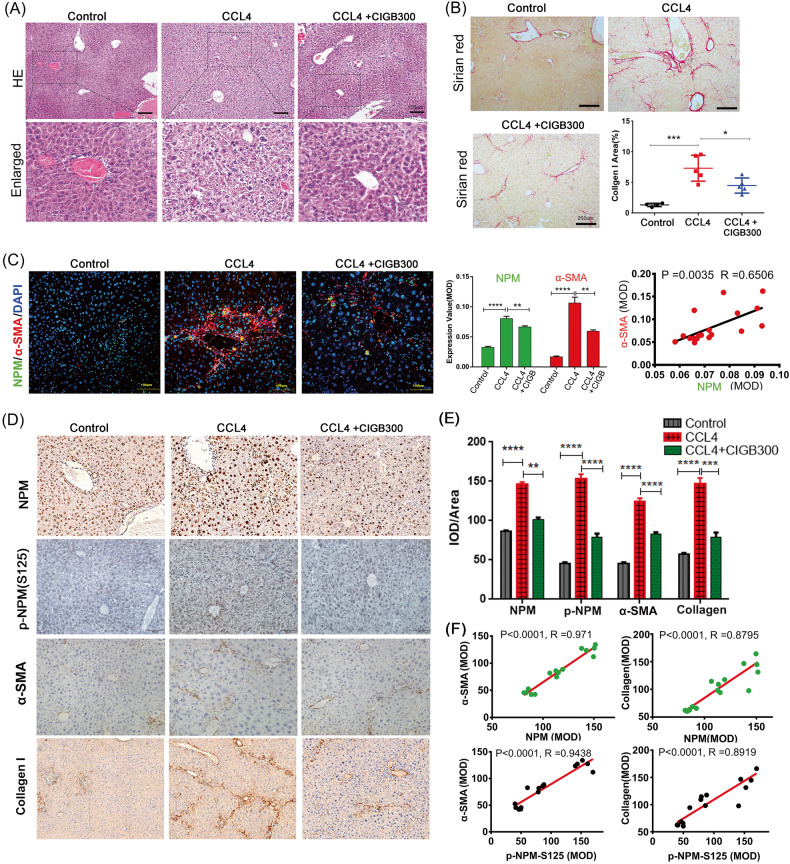
NPM inhibitor CIGB300 significantly inhibited liver fibrosis in mice. Liver fibrosis was induced by intraperitoneal injection of CCl_4_ in C57BL/6 mice, and CIGB300 was dissolved in PBS and administered via tail vein to inhibit the function of NPM protein. Five mice were placed in each group. **A** Representative images of HE staining showed the pathology of liver tissue in mouse models of liver fibrosis induced by CCL_4_.CIGB300 significantly protected liver tissue from damage caused by CCl_4_ to liver cells and tissue structures. Scale bar corresponds to 100 μm. **B** Sirius red staining showed significant deposition of collagen in the liver tissues of mice induced by CCl_4_, which significantly decreased after treatment with CIGB300 (*n* = 5/group). Scale bar corresponds to 250 μm. **C** Immunofluorescence of paraffin sections showed that both NPM and α-SMA expression levels were increased in the liver tissues of CCl_4_-induced mice. CIGB300 treatment significantly reduced both. The expression level of NPM and α-SMA was positively correlated. NPM was labeled with green fluorescence, and α-SMA was labeled with red fluorescence. Representative images. Scale bar corresponds to 50 μm. **D, E** IHC detection showed that CIGB300 significantly inhibited CCl_4_-induced upregulation of NPM protein, NPM S125 phosphorylation, α-SMA, and collagen I protein in mouse fibrosis liver tissue. **F** Analysis of quantitative data showed a positive correlation between NPM protein or NPM S125 phosphorylation and liver fibrosis markers in the liver tissues. Data shown are representatives of three independent experiments. *n* ≥ 3; mean ± SEM; *p < 0.05, ***p < 0.001; *T* test.

HE and Sirius red staining were used to observe the pathological changes and collagen deposition of liver tissue, respectively. CCl_4_ causes liver damage via the destruction or disappearance of normal hepatic lobules, which are replaced by false lobules, hepatic cell edema, steatosis, and nodular regeneration arrangement. CIGB300 significantly alleviate these histological changes (Fig. 4A). Sirius red staining showed that CIGB300 significantly reduced abnormal collagen deposition and fibrosis caused by CCl_4_ (Fig. 4B). IHC analysis showed that the phosphorylation of NPM (Ser125), which was significantly up-regulated in liver fibrosis tissues, was down-regulated by CIGB300 (Fig. 4D, E). Immunofluorescence (Fig. 4C) and IHC analysis (Fig. 4D, E) confirmed that NPM, α-SMA, and collagen I, which also are up-regulated by CCl_4_, were down-regulated by CIGB300.Correlation analysis showed that NPM was positively correlated with α-SMA and collagen I, and the phosphorylation of NPM (Ser125) was positively correlated with these liver fibrosis markers (Fig. 4F). Interestingly, CIGB300 not only inhibited Ser125 phosphorylation, but also reduced NPM in tissues (Fig. 4D), suggesting that the expression and phosphorylation of NPM may be regulated in a complex way in liver tissue. The downstream molecules regulated by NPM may interact with different cells in liver tissues in a positive feedback manner, resulting in the change of NPM expression. It is important to note that CIGB300 targets are not cell specific, which means that the function involved in NPM Ser125 may be inhibited in all types of liver tissue cells.

Therefore, the NPM in mouse liver tissue was positively correlated with the α-SMA in HSCs, and the inhibition of NPM protein function of liver tissue cells can reduce the activated HSCs and collagen secretion, thereby inhibiting liver fibrosis.

### NPM promotes liver fibrosis by inhibiting the apoptosis of HSCs through the Akt/ROS/Apoptosis pathway

**Fig. 5:**
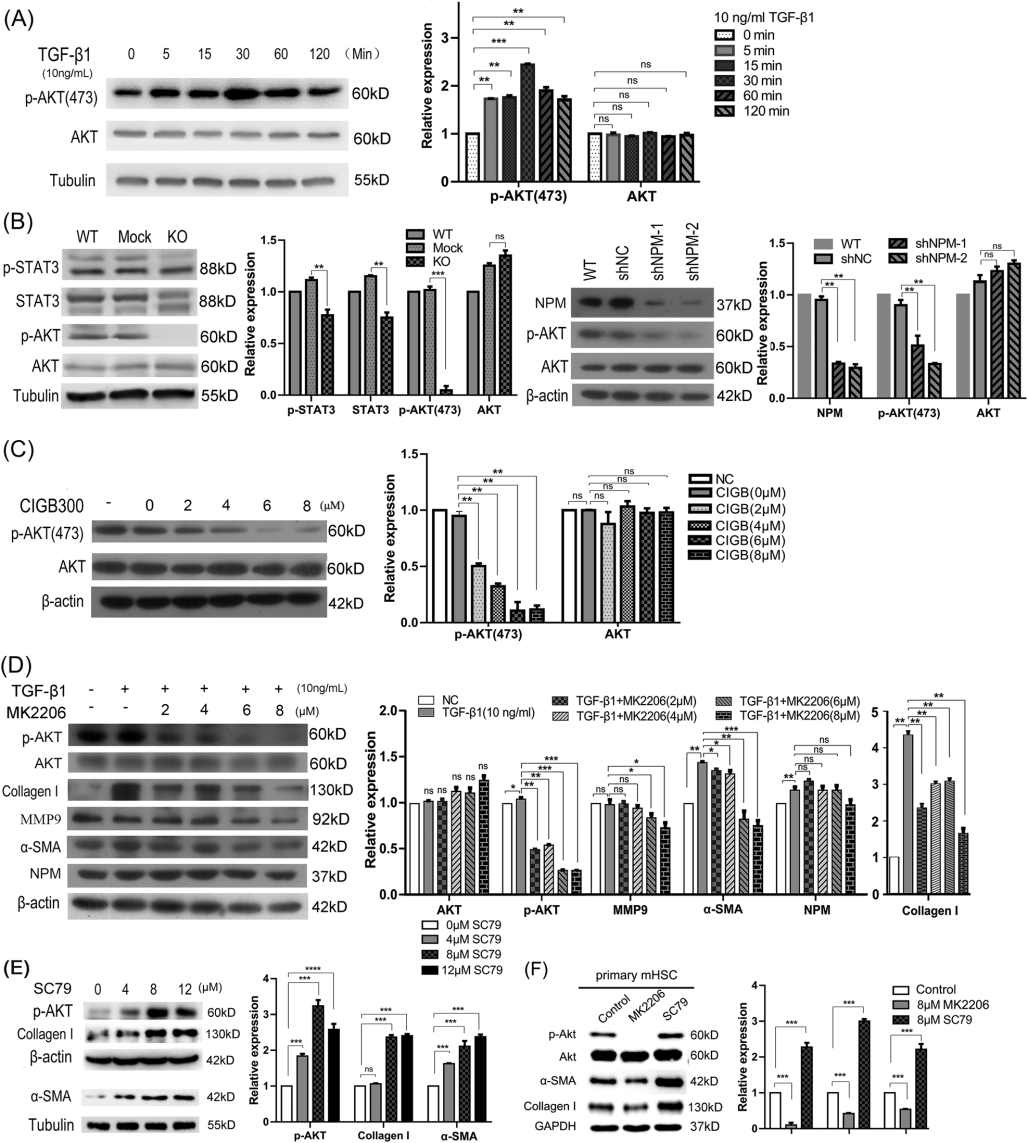
NPM regulated Akt phosphorylation and Akt promoted liver fibrosis markers. **A** Fibrogenic factor TGF-β1 up-regulated Akt phosphorylation in LX-2 cells. **B** NPM knockout or knockdown inhibited AKT phosphorylation in hepatic stellate cells LX-2. The knockout efficiency of NPM in LX-2 has been verified and shown in Fig. 2A. **C** The NPM inhibitor CIGB300 inhibited AKT phosphorylation in HSCs LX-2. **D** Akt phosphorylation inhibitor MK2206 reduced the protein expression of collagen I, MMP9, and α-SMA in LX-2 cells. **E** Akt activator SC79 up-regulated the expression of markers of liver fibrosis. **F** The collagen I and α-SMA were decreased or increased by Akt inhibitor or activator correspondingly in primary hepatic stellate cells. Data shown are representatives of three independent experiments. *n* ≥ 3; mean ± SEM; *p < 0.05, ***p < 0.001; *T* test.

The in vitro and in vivo studies mentioned above confirmed the role of NPM in promoting liver fibrosis. The mechanism in which NPM promotes liver fibrosis was investigated in HSCs. The active nuclear Akt interacts with NPM to prevent the proteolytic cleavage of NPM [29], but whether NPM affects Akt activity remains unclear. Western blot assay confirmed that Akt phosphorylation in HSCs was up-regulated by fibrogenic factor TGF-β1 (Fig. 5A) but was significantly inhibited by NPM silencing (Fig. 5B) or CIGB300 treatment (Fig. 5C). Therefore, NPM may promote liver fibrosis by activating Akt pathway. To test this hypothesis, MK2206, an inhibitor of Akt, was used to treat HSCs. Results show that the liver fibrosis markers α-SMA and collagen I were downregulated when Akt phosphorylation was inhibited under different concentrations of MK2206 (Fig. 5D). While Akt activator SC79 up-regulated α-SMA and collagen I in LX-2 cells (Fig. 5E). Similar results were confirmed in the primary hepatic stellate cells (Fig. 5F). Therefore, NPM promotes liver fibrosis by activating Akt signaling.

The activation of HSCs is the central event in the process of liver fibrosis, and ROS is an important effector medium at the initial stage of liver fibrosis, which can activate quiescent HSCs [30]. However, different levels of ROS have different effects on cells [31]. Moderate ROS inactivates PTEN, promotes the PI3K/Akt signaling pathway, and ultimately leads to tumor progression [32]. Excessive ROS can damage the cellular structure of proliferative cancer cells, especially excessive ROS induces DNA mutations and impair genomic integrity, leading to cell senescence and death [33]. Our previous studies confirmed that NPM downregulates ROS by antioxidant protein PRDX6 in tumor cells [34, 35]. Therefore, NPM may have a protective effect on the activated proliferative HSCs by downregulating ROS.

**Fig. 6:**
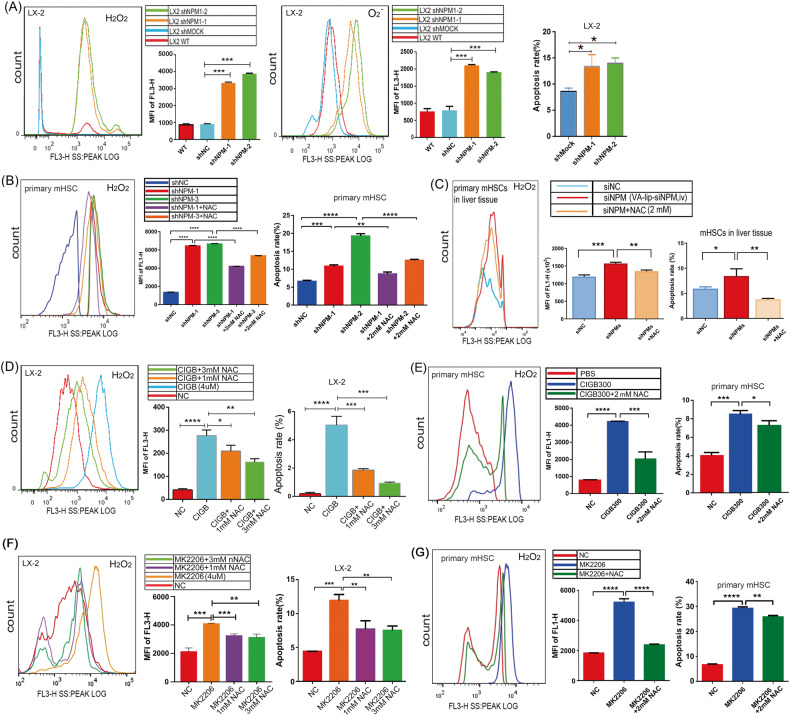
NPM downregulated ROS and reduced the apoptosis of HSCs via the Akt pathway. CellROX Orange reagent and dihydroethidium were used to label intracellular hydrogen peroxide and superoxide anion. The ROS level and apoptosis of HSCs were detected by flow cytometry. **A** Flow cytometry results showed that NPM knockdown remarkably reduced hydrogen peroxide and superoxide anion in human LX-2 cells and primary mouse HSCs. **B** Flow cytometry showed NPM knockdown significantly induced the apoptosis of LX-2 and primary mouse HSCs. **C** Flow cytometry analysis of primary HSCS isolated from liver tissue of fibrotic mice showed that when VA-lip-siNPM specifically down-regulated the NPM expression of HSC in liver tissue, ROS was up-regulated and apoptosis was increased. When the up-regulated ROS in liver tissue was down-regulated by NAC, the apoptosis of HSC was reduced (*n* = 3). **D** Flow cytometry results showed that the ROS inhibitor NAC dose-dependently inhibited CIGB-induced hydrogen peroxide and apoptosis in LX-2 cells. **E** NAC inhibited CIGB-induced hydrogen peroxide and apoptosis in primary mouse HSCs. **F** Flow cytometry results showed that NAC reduced MK2206-induced hydrogen peroxide and reduced apoptosis in LX-2 cells. **G** NAC reduced MK2206-induced hydrogen peroxide and reduced apoptosis in primary mouse HSCs. The data shown are representative of three independent experiments and quantification data were presented as mean ± SEM; MFI mean fluorescence intensity, WT untreated cells, NC negative control, ns non-significant difference, **p < 0.01, ***p < 0.001; *T* test.

To confirm above hypothesis, we examined the effects of NPM on ROS and apoptosis in human LX-2 cells and primary mouse HSCs. The results of flow cytometry showed that after NPM knockdown in human LX-2 cells and primary mouse HSCs, intracellular hydrogen peroxide and superoxide anion were significantly upregulatedand apoptosis was induced (Fig. 6A, B, SFig. 4A). To verify in vivo that NPM could decrease ROS and reduce apoptosis of HSCs, we repeated the mouse liver fibrosis model experiment in Fig. 3, using VA-lip-siNPM to reduce NPM expression of mHSC in liver tissue and NAC to inhibit ROS. We developed a simultaneous and rapid separation and labeling method (about 110 min in total) to label the ROS status and apoptosis level of primary HSCs during isolation for subsequent flow cytometry detection. WB experiments were performed with the remaining primary hepatic stellate cells after flow test, and it was confirmed that the expression of NPM was effectively knocked down by VA-lip-siNPM. The results of flow cytometry confirmed that NPM Knockdown in HSCs up-regulated ROS and induced more apoptosis in mouse hepatic fibrosis models, while inhibition of ROS by NAC reduced siNPM-induced apoptosis (SFig. 4B, Fig. 6C). NPM inhibitor CIGB300 also increased ROS levels and significantly increased apoptosis in LX-2 cells (Fig. 6D). ROS inhibitor N-Acetyl-L-cysteine (NAC) decreased CIGB300-induced ROS and reduced CIGB300-induced apoptosis in LX-2 cells and primary mHSCs (Fig. 6D, E, SFig. 5). Therefore, NPM inhibits apoptosis by downregulating ROS level, while CIGB300 promotes the apoptosis of HSCs by upregulating ROS.

Fibrosis regression is associated with the inactivation or apoptosis of HSCs and myofibroblasts, making death of HSCs an important mechanism for the resolution of liver fibrosis [36]. The knockdown and inhibition of NPM both inactivate Akt signaling and increase ROS and apoptosis. We further analyzed the effects of Akt on ROS and apoptosis in HSCs. Flow cytometry results showed that Akt inhibitor MK2206 significantly increased the ROS level and induced the apoptosis, while ROS inhibitor NAC reduced MK2276-induced ROS and significantly reduced apoptosis in LX-2 cells (Fig. 6F) and primary mHSCs (Fig. 6G), confirming the existence of the AKT/ROS-induced apoptosis axis in HSCs.

Summarizing the above results, it can be concluded that NPM promotes liver fibrosis by inhibiting the apoptosis of HSCs through the Akt/ROS/Apoptosis pathway.

### LncMIAT up-regulated by Akt promotes the liver fibrosis markers by upregulating TGF-β2 by competitively sponging miR-16-5p

In addition to the Akt/ROS/Apoptosis pathway, we attempted to find other pathways involved in the fibrotic effects of NPM. In transcriptome data analyzed using DESeq2 software, we found that long non-coding RNA molecule MIAT was the only down-regulated gene with significant differences after treatment with Akt inhibitor MK2206 (SFig. 6A), and MIAT has been shown to be involved in the development of renal fibrosis [37]. We wondered if MIAT was involved in the regulation of liver fibrosis. Therefore, we detected MIAT expression and its pro-fibrosis effect in hepatic fibrosis cell models.

**Fig. 7:**
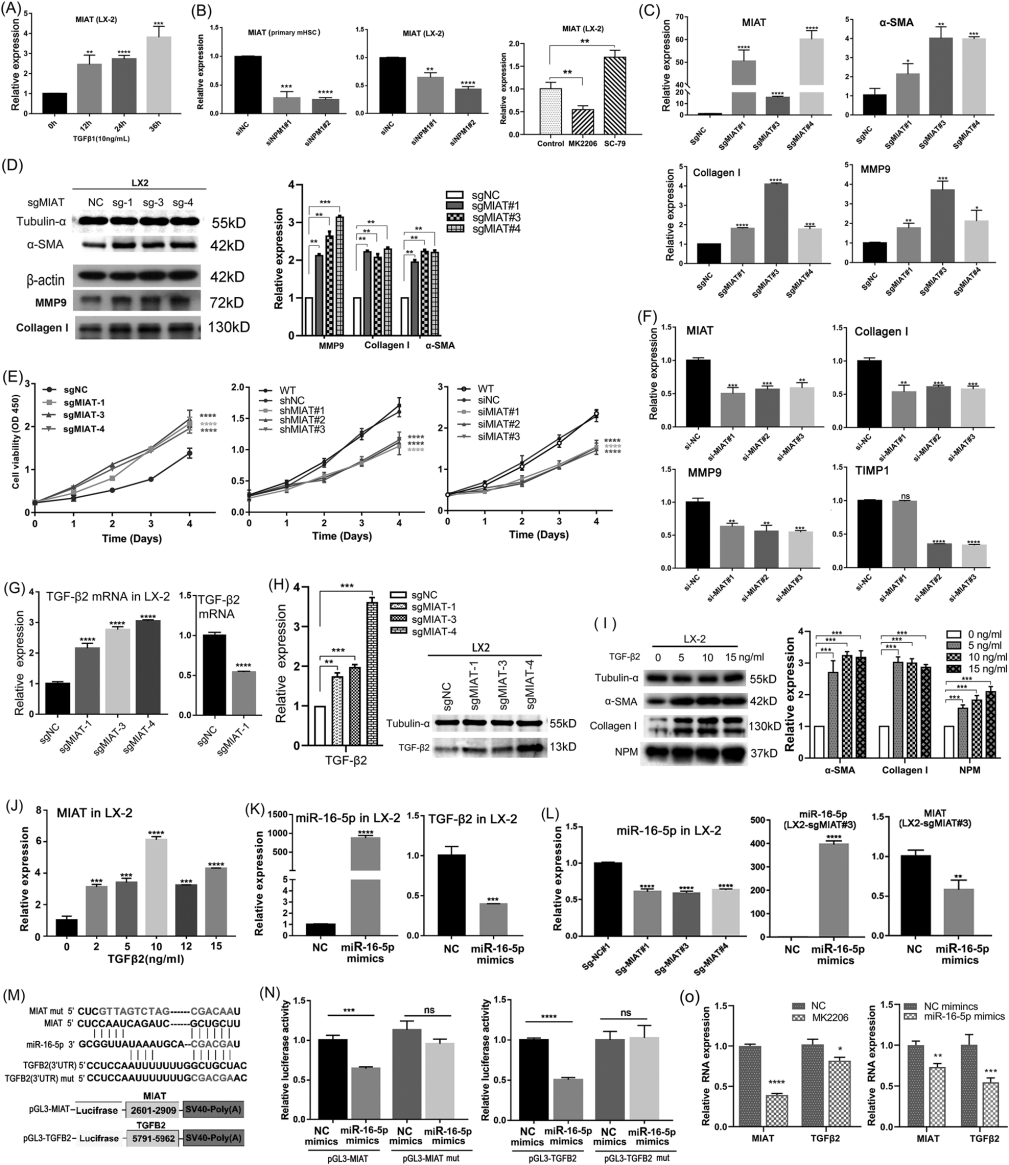
LncRNA MIAT, which was up-regulated by NPM, promotes liver fibrosis markers by regulating TGF-β2 mRNA by competitively sponging miR-16-5p. **A** Quantitative PCR showed that the expression of MIAT was up-regulated in TGFβ1-treated LX-2 cells. **B** Quantitative PCR showed that the expression of NPM was knocked down by small interfering RNA, and the level of MIAT was significantly downregulated in LX-2 and LO2 cells. **C** Quantitative PCR showed that the endogenous overexpression of MIAT up-regulated the expression levels of α-SMA, collagen, and MMP9. The endogenous expression of MIAT was up-regulated by CRISPR/dCAS9 gene activation system. The sgMIAT is a small guide RNA targeting the promoter region of MIAT. Inactivated dCas9 binds to the promoter, and the fused transcriptional activating element on dCas9 remarkably up-regulated the endogenous expression of MIAT. **D** Western blot results confirmed the up-regulated expression of α-SMA, collagen I, and MMP9 and showed that MIAT increased the level of cyclin D1. **E** CCK8 proliferation assay showed that the proliferation ability of LX-2 cells was up- or downregulated after MIAT overexpression or shRNA/siRNA deletion. **F** Quantitative PCR showed that collagen I and MMP9 expression levels remarkably decreased after siRNA knockdown of MIAT expression. **G** Quantitative PCR analysis showed that endogenous MIAT overexpression up-regulated TGF-β2 mRNA, and MIAT knockdown decreased TGF-β2 mRNA. **H** Western blot analysis results confirmed that activated MIAT expression up-regulated TGF-β2 and α-SMA in LX-2 cells. **I** Western blot analysis results showed that TGF-β2 treatment increased the expression of collagen I and α-SMA in LX-2 cells. **J** Quantitative PCR analysis showed TGF-β2 up-regulated MIAT in LX-2 cells. **K** Transfection of LX-2 cells with miR-16-5p mimics reduced the expression of TGF-β2. **L** Activation of endogenous MIAT expression in LX-2 cells reduced the expression of miR-16-5p, while transfection of miR-16-5p mimics LX-2 cells, and activated MIAT decreased the endogenous MIAT. **M** miR-16-5p can bind to sequences on MIAT and TGF-Β2 RNA molecules. The sequences between bases 2601–2909 on MIAT and 5791–5962 on TGF-β2 mRNA were inserted into the downstream sites of the luciferase coding region of PGL3 plasmid. **N** Dual-luciferase reporter assay showed that miR-16-5p mimics reduced luciferase expression, but mimics had no binding effect on the mutated binding sites on MIAT or TGF-β2. **O** qPCR analysis of primary HSCs isolated from liver tissues of fibrotic mice showed that Akt inhibitor MK2206 inhibited the expression of MIAT and TGF-β2 in vivo, while miR-16-5p mimics also down-regulated the expression of MIAT and TGF-β2 in mHSC in vivo. The data shown are representatives of three independent experiments. *n* ≥ 3; mean ± SEM; *p < 0.05, **p < 0.01, ***p < 0.001; *T* test.

MIAT significantly increased in LX-2 cells treated with TGF-β1 (Fig. 7A). We further investigated the regulatory relationship between NPM, Akt, and MIAT, and found that NPM knockdown by siRNA significantly decreased MIAT expression in LX-2 cells and primary mouse hepatic stellate cells, while Akt inhibitor MK2206 or activator SC79 down-regulated or up-regulated MIAT expression (Fig. 7B), respectively. Combined with the previously confirmed role of NPM in regulating Akt, these results confirm that NPM regulates the expression of MIAT through Akt signaling.

Next, we further studied the effect of MIAT on liver fibrosis markers. The CRISPR-dCas9 system was used to activate the expression of endogenous MIAT (Fig. 7C), and the up-regulated MIAT significantly increased the hepatic fibrosis markers α-SMA, collagen I, and MMP9 of LX-2 (Fig. 7C, D) and promoted cell proliferation (Fig. 7E). MIAT knockdown decreased the proliferation (Fig. 7E) and downregulated liver fibrosis markers (Fig. 7F). Therefore, we have identified another possible regulatory pathway for NPM to promote fibrosis through Akt/MIAT.

To determine the downstream fibrosis-related genes regulated by MIAT, RNA-seq analysis was performed on the LX-2 cells with down-regulated or up-regulated MIAT. Among the most differentially expressed genes, TGF-β2 was up-regulated by MIAT (SFig. 6B). The upregulation effect of MIAT on TGF-β2 was confirmed by quantitative PCR and Western blot in LX-2 cells with activated MIAT expression (Fig. 7G, H). MIAT knockdown reduced TGF-β2 mRNA in HSCs LX-2 (Fig. 7G). Further test showed that α-SMA and collagen I were significantly up-regulated by TGF-β2 in LX-2 (Fig. 7I). Therefore, MIAT can regulate the secretion of TGF-β2 and promote the expression of liver fibrosis markers. Interestingly, TGF-β2 also up-regulated the MIAT level of HSCs, creating a positive feedback (Fig. 7J).

In order to elucidate the molecular mechanism by which MIAT regulates TGF-β2 levels, sequence analysis using the DIANA tool revealed that miR-16-5p had multiple binding sites on MIAT RNA and TGF-β2 mRNA (SFig. 7), suggesting that miR-16-5p might regulate the intracellular levels of MIAT and NPM as a competing endogenous RNA. To prove this hypothesis, we transfected miR-16-5p mimics into LX-2 cells, and the intracellular MIAT and TGF-β2mRNA were significantly downregulated (Fig. 7K, L). The miR-16-5p mimics also downregulated the type I and III collagen and the cell proliferation ability of LX-2 (SFig. 8). Four nucleotide fragments around the miR-16-5p binding sites on MIAT molecule were cloned and inserted into the 3′ UTR of the luciferase of the pGL3 plasmid. The mutated fragment was constructed as a control (Fig. 7M, SFig. 9). Double luciferase reporter assay revealed that miR-16-5p mimics could bind to one of the MIAT fragments (2601–2909) and significantly reduce luciferase expression but not to the mutated fragment (Fig. 7N). Therefore, miR-16-5p can bind to MIAT to regulate its stability. We further inserted the miR-16-5p binding fragment of TGF-β2 mRNA into the 3′ UTR of luciferase to construct pGL3-TGF-β2 and control plasmid with mutated site. Dual-luciferase reporter assay confirmed that miR-16-5p could bind TGF-β2 mRNA and regulate its level (Fig. 7N).

To validate the Akt-MIAT-miR-16-TGF-β2 pathway in vivo, we treated C57 mice with hepatic fibrosis with an AKT inhibitor, MK2206, to analyze the effect of Akt on downstream MIAT and TGF-β2 gene expression in HSCs. We further used miR-16-5p mimics to treat mice with liver fibrosis and analyzed the effects of miR-16-5p on MIAT and TGF-β2. After 36 h of treatment, mice in control group and each treatment group were killed, and HSCs were isolated from liver tissues. The expression changes of MIAT and TGF-β2 were detected in the isolated primary HSCs of each group, so as to verify the influence of AKT signal on MIAT and TGF-β2, as well as the promotion effect of MIAT on TGF-β2 through adsorption of miR-16-5p. The qPCR results showed that inhibition of Akt signal down-regulated MIAT RNA and TGF-β2 mRNA, and the mimics of miR-16-5p can simultaneously down-regulate MIAT and TGF-β2 in vivo (Fig. 7O). These results suggest that Akt promotes the expression of MIAT, and the up-regulated MIAT in turn up-regulates TGF-β2 mRNA by competitively sponging miR-16-5p, thus promoting the expression of liver fibrosis markers of HSCs in an autocrine manner.

### NPM promotes TGF-β2 secretion in hepatocytes and Kupffer cells, which in turn activates HSCs

**Fig. 8:**
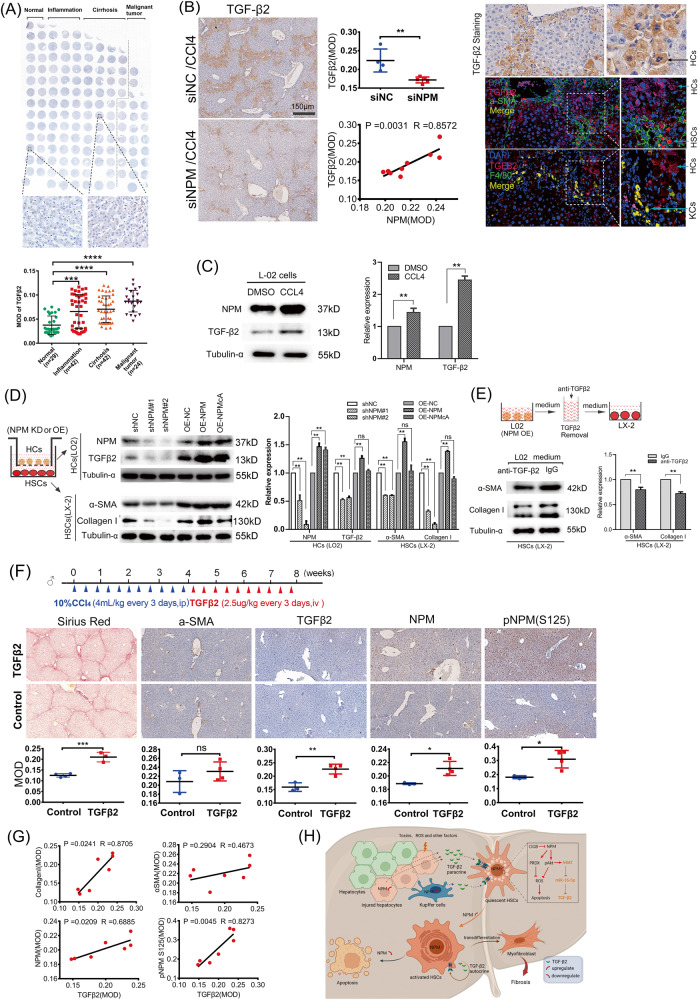
NPM promotes the secretion of TGF-β2 and regulates the activation of hepatic stellate cells. **A** Analysis of human liver disease tissue arrays (LV20812a, trial) showed that TGF-β2 is up-regulated in fibrotic tissues. **B** IHC staining and immunofluorescence analysis showed that TGF-β2 is mainly distributed in liver cells and macrophages. **C** Western blot confirmed that the necrotic hepatocytes induced by CCl_4_ expressed more NPM and TGF-β2 proteins. Cells were treated with 10 mmol/mL CCl_4_ dissolved in DMSO for 6 h, and cells were collected for protein extraction after 24 h. **D** Western blot analysis confirmed that NPM knockdown in LO2 cells significantly downregulated TGF-β2 expression, and the expression of collagen I and α-SMA in the LX-2 cells co-cultured with LO2 decreased. **E** Western blot analysis results confirmed that the expression of markers of liver fibrosis in LX-2 cells was downregulated when the TGF-β2 secreted by LO2 cells in culture medium was removed with antibody. **F** In the mice model, TGF-β2 was injected into CCl_4_-induced hepatic fibrosis mice by tail vein during recovery. Sirius red staining and IHC analysis showed that TGF-β2 treatment up-regulated the expression of markers of liver fibrosis and NPM. **G** Correlation analysis confirmed that TGF-β2 was strongly correlated with NPM and collagen deposition. **H** Working model of NPM in promoting liver fibrosis. The data shown are representatives of three independent experiments. Mean ± SEM; *p < 0.05, **p < 0.01, ***p < 0.001; *T* test.

Our results confirmed the existence of an NPM/MIAT/miR-16-5p/TGF-β2 regulatory pathway in human HSCs. IHC staining of human liver disease tissue arrays showed that TGF-β2 was up-regulated in fibrotic tissues (Fig. 8A). IHC staining indicated that TGF-β2 protein was significantly down-regulated in fibrotic tissues of siNPM-treated mice, and the expression of TGF-β2 was strongly correlated with that of NPM (*P* = 0.003, *R* = 0.8572). TGF-β2 was mainly expressed in the cells of the portal tract and injured hepatocytes, and the latter showed the characteristic nuclear pyknosis of necrotic cells (Fig. 8B, SFig. 2). Further co-location analysis by immunofluorescence revealed that TGF-β2 was mainly expressed in necrotic hepatocytes and Kupffer cells rather than HSCs in fibrotic liver tissues (Fig. 8B).

Considering that hepatocytes represent the largest proportion of cells in liver tissue, necrotic hepatocytes may express much more TGF-β2 protein than those from HSCs or from hepatic macrophages. To confirm the upregulation of TGF-β2 protein in necrotic apoptotic hepatocytes, we induced the necrosis of cultured human hepatocyte cell line LO2 by using CCl_4_. Western blot assay confirmed that the NPM and TGF-β2 protein both increased after 6 h of CCl_4_ treatment (Fig. 8C).

Whether NPM in hepatocytes promotes the secretion of TGF-β2 through paracrine, thus affecting the activation of HSCs and the expression of fibrosis markers, need to be determined. Accordingly, we knocked down or overexpressed NPM in hepatocytes LO2, and Western blot analysis confirmed that TGF-β2 expression remarkably changed. Moreover, the markers of liver fibrosis in LX-2 cells co-cultured with LO2 cells in Transwell chambers were down- or up-regulated (Fig. 8D). To further confirm the role of TGF-β2 in the co-culture system, we removed TGF-β2 protein from the culture medium via immunoprecipitation by using the TGF-β2 antibody, and WB showed significantly reduced α-SMA and collagen I expression in LX-2 cells cultured in this conditioned medium (Fig. 8E). Therefore, NPM can activate the hepatocyte secretion of TGF-β2, thus promoting HSC activation and collagen synthesis.

TGF-β2 was strongly expressed in macrophages in the portal vein region, and the expression intensity was much higher than in hepatocytes or HSCs (Fig. 8B, SFig. 3). To confirm the effect of NPM on TGF-β2 expression in macrophages, we knocked down or overexpressed NPM protein in human monocyte THP-1-derived macrophages and mouse macrophages RAW264.7. WB results confirmed that TGF-β2 protein was regulated by changes in NPM expression. Moreover, the expression of α-SMA and collagen I changed in human HSCs (LX-2) or mouse HSCs (SFig. 10A, B) co-cultured with macrophages. To confirm that the changes of α-SMA and collagen I are caused by TGF-β2 protein, we further removed TGF-β2 protein from macrophage culture medium supernatant via immunoprecipitation. The expression of α-SMA and collagen I in LX-2 or mouse HSCs changed significantly after 36 h of culture in this conditioned medium (SFig. 10C), indicating that the TGF-β2 secreted by macrophages remarkably affected the activation and collagen expression of HSC in the co-culture system.

### TGF-β2 promotes CCl_4_-induced liver fibrosis

TGF-β2 is involved in the fibrosis of biliary-derived liver diseases [16]. The effect of TGF-β2 on CCl_4_-induced liver fibrosis in Balb/c mice was determined by tail vein injection of active TGF-β2 for 4 weeks after CCl_4_ induction (Fig. 8F). IHC staining (Fig. 8F) and Western blot (SFig. 11A) analysis showed that TGF-β2 increased the expression of collagen I, NPM, and phosphorylated AKT. CCl_4_-induced liver fibrosis in C57Bl6J mice showed similar results (SFig. 11B). Correlation analysis confirmed that TGF-β2 was strongly correlated with NPM and collagen deposition (Fig. 8G). Therefore, in combination with previous results of hepatocytes and macrophage, our findings confirmed that NPM promoted the secretion of TGF-β2 in hepatocytes and macrophages, activated HSCs in a paracrine manner, and promoted hepatic fibrosis.

### Working model

In summary, we proposed a working model to demonstrate the mechanism in which NPM promotes liver fibrosis (Fig. 8H); NPM, which is widely expressed in liver tissue, is up-regulated during liver fibrosis by various factors. The upregulation of NPM in HSCs further inhibits ROS levels and ROS-induced apoptosis by activating Akt and by regulating PRDX6 expression, thereby promoting HSC activation and collagen secretion. CIGB300, an inhibitor of NPM phosphorylation at the Ser125, inhibits Akt and upregulates the apoptosis of HSCs, thereby inhibiting the progression of hepatic fibrosis. Moreover, NPM upregulates the non-coding RNA molecule MIAT by Akt, thus promoting the expression and secretion of TGF-β2 by competitively sponging miR-16-5p. TGF-β2, which was derived from injured hepatocytes, Kupffer cells, and HSCs, activates the proliferation and collagen secretion of HSCs by paracrine or autocrine modes and promotes the process of liver fibrosis. Considering the large number of hepatocytes, injured hepatocytes may be the main source of TGF-β2 in the early stage of hepatic fibrosis in mice.

## Discussion

The relevance between NPM and liver fibrosis has not been reported. In the present study, we demonstrated that NPM silencing or NPM inhibitor CIGB300 significantly alleviated liver fibrosis in both animal and cellular models, indicating that NPM promotes liver fibrosis.

Liver fibrosis is regulated by various parenchymal and non-parenchymal cells in liver. Immunostaining showed that NPM was expressed in hepatocytes, cholangiocytes, as well as in various cells of portal vein region, including HSCs and Kupffer cells. Especially in CCl4-treated mouse liver, NPM was strongly expressed in the cytoplasm of injured hepatocytes that surround the portal region, suggesting NPM is not limited to HSCs to play a pro-fibrotic role.

Mechanistic analysis showed that NPM may regulate liver fibrosis through the Akt/ROS axis of HSCs. Akt signaling regulates cell proliferation and survival and plays a key role in the activation and recruitment of inflammatory cells, thereby activating HSCs to promote liver fibrosis [38, 39]. Nuclear Akt interacts with NPM and protects NPM from proteolytic cleavage, thus enhancing cell survival [29]. Our results confirmed that NPM in turn promotes Akt phosphorylation. The effect of Akt in HSCs was further determined, and the results show that the inhibition of Akt signaling leads to an increase in ROS level, which promoted HSC apoptosis. Moreover, NPM knockdown or inhibition in HSCs remarkably increased ROS levels, which resulted in more apoptosis, confirming the NPM/Akt/ROS apoptosis axis. Besides, we have confirmed that NPM inhibited the excessive ROS by upregulation of the antioxidant protein PRDX6, thus protecting cells from apoptosis [34, 35]. Moderate ROS can activate HSCs, but excessive ROS can promote apoptosis [5, 40]. Therefore, NPM decreased ROS in HSCs by activating Akt or by upregulating PRDX6, thus protecting the activated HSCs from apoptosis and promoting liver fibrosis.

It should be noted that the exact mechanism by which NPM regulates Akt signaling is not yet known, but previous studies have provided some clues. Studies have shown that NPM protein directly binds to the PH domain of phosphorylated AKT protein, and this interaction is crucial for the anti-apoptotic effect of NPM [29]. In addition, PI(3,4,5)P3 on the membrane is known to bind to the PH domain of AKT to promote phosphorylation of AKT and prevent its dephosphorylation [41]. Considering that NPM has both nuclear and nuclear localization signals and can travel between cytoplasm and nucleus, and is mainly localized in the nucleolus. Moreover, nuclear PI(3,4,5)P3 competes with Akt to preferentially bind NPM [42]. Therefore, we speculate that NPM may promote AKT signal transduction by influencing the nucleation process, stability and distribution of phosphorylated AKT in the nucleus. On the one hand, cytoplasmic NPM binds to the PH domain of AKT to promote the nucleation of phosphorylated AKT and may also promote the stability of phosphorylated AKT. On the other hand, incoming NPM enriches phosphorylated AKT at specific sites through its localization in the nucleolus, thereby promoting AKT’s action on specific substrates. Akt localization in the nucleus helps to limit its substrate specificity and induce context-specific cellular responses [41]. The above hypothesis still needs a lot of experimental evidence to verify its authenticity.

The role of NPM in hepatic fibrosis is much more complex than the NPM/Akt/ROS/apoptosis axis in HSCs. In the results, the AKT’s phosphorylation is wholly blocked while the markers of liver fibrosis change are mild, which indicates other factors are playing a role. Based on the similar distribution of NPM protein and TGF-β2 protein in hepatic fibrosis tissue, we further investigated the regulatory relationship between NPM and TGF-β2, the source of TGF-β2 protein in liver tissues, and its effect on liver fibrosis. Mechanistic analysis confirmed that NPM up-regulated MIAT, which promoted TGF-β2 expression by competitively sponging miR-16-5p. Immunostaining analysis showed that TGF-β2 was mainly expressed in Kupffer cells, hepatocytes, and HSCs. Kupffer cells showed the highest staining intensity of TGF-β2. However, considering the numerical superiority of hepatocytes, TGF-β2 in liver are likely to be derived mainly from the injured hepatocytes. In addition, our mice model results suggest that TGF-β2 promotes CCl_4_-induced fibrosis. Previous studies also confirmed that TGF-β2 silencing inhibited biliary-derived liver fibrosis [16]. Therefore, as another pro-fibrotic pathway of NPM in Kupffer cells, hepatocytes or HSCs, NPM promotes the expression and secretion of TGF-β2, thus activating HSCs in a paracrine or autocrine manner.

Notably, NPM promotes liver fibrosis, but not all NPM inhibitors can reduce liver fibrosis. CIGB300, which binds to NPM Ser125 and inhibits its phosphorylation, inhibited liver fibrosis by down-regulating Akt phosphorylation. But another NPM oligomerization inhibitor NSC348884 [28], whose mechanism of NPM inhibition is still debated [43, 44], did not significantly inhibit liver fibrosis in mice duo to its excessive proapoptotic effect on hepatocytes besides on HSCs (data not shown). Therefore, due to the importance of NPM in cell proliferation and apoptosis, the possible side effects of NPM inhibition should be considered in the design of drugs targeting NPM to inhibit liver fibrosis.

It should be noted that, the methodology used in this study shows relevant restraints. CCl_4_-induced fibrosis is an artificial model that is standardized and easy to use, which may not be sufficient to fully elucidate the mechanism in which NPM promotes fibrosis. Indeed, no significant biliary response was observed in CCl_4_-induced hepatic fibrosis, but significant biliary reaction and positive expression of NPM protein was observed in biliary epithelial cells on human liver disease microarrays (data not shown), suggesting that bile epithelial cells may also be an important source of TGF-β2. In addition, another methodological restraint is the lack of conditional knockout mice to restrict effects to distinct cell types in the liver, which is attributed to the fact that no conditional knockout mice have yet been established for HSCs. Therefore, the mechanism obtained in the present study is limited to toxin-induced liver fibrosis, and the pro-fibrotic mechanism of NPM needs to be further studied using a biliary fibrosis model in conditional knockout mice.

Taken together, our results demonstrate that NPM promotes liver fibrogenesis by inhibiting the Akt/ROS-induced apoptosis in HSCs and upregulating Akt/MIAT-induced TGF-β2 in hepatocytes, Kupffer cells and HSCs. Inhibition of NPM or application of NPM inhibitor remarkably attenuated liver fibrosis. NPM serves a potential new drug target for liver fibrosis.

## Supplementary information

Supplementary material (Figures and tables)

Original data files

Reproducibility checklist

## Data Availability

All data generated or analyzed during this study are available from the corresponding author on reasonable request.
