# Supplementary material for: NPM promotes hepatotoxin-induced fibrosis by inhibiting ROS-induced apoptosis of hepatic stellate cells and upregulating lncMIAT-induced TGF-β2
Source: Cell Death Dis. 2023 Aug 30;14(8):575. doi: 10.1038/s41419-023-06043-0 (PMC10469196; doi:10.1038/s41419-023-06043-0)
Supplement: Supplementary file 1 — Supplementary material (Figures and tables) [file 41419_2023_6043_MOESM1_ESM.docx]

**Supplementary material**


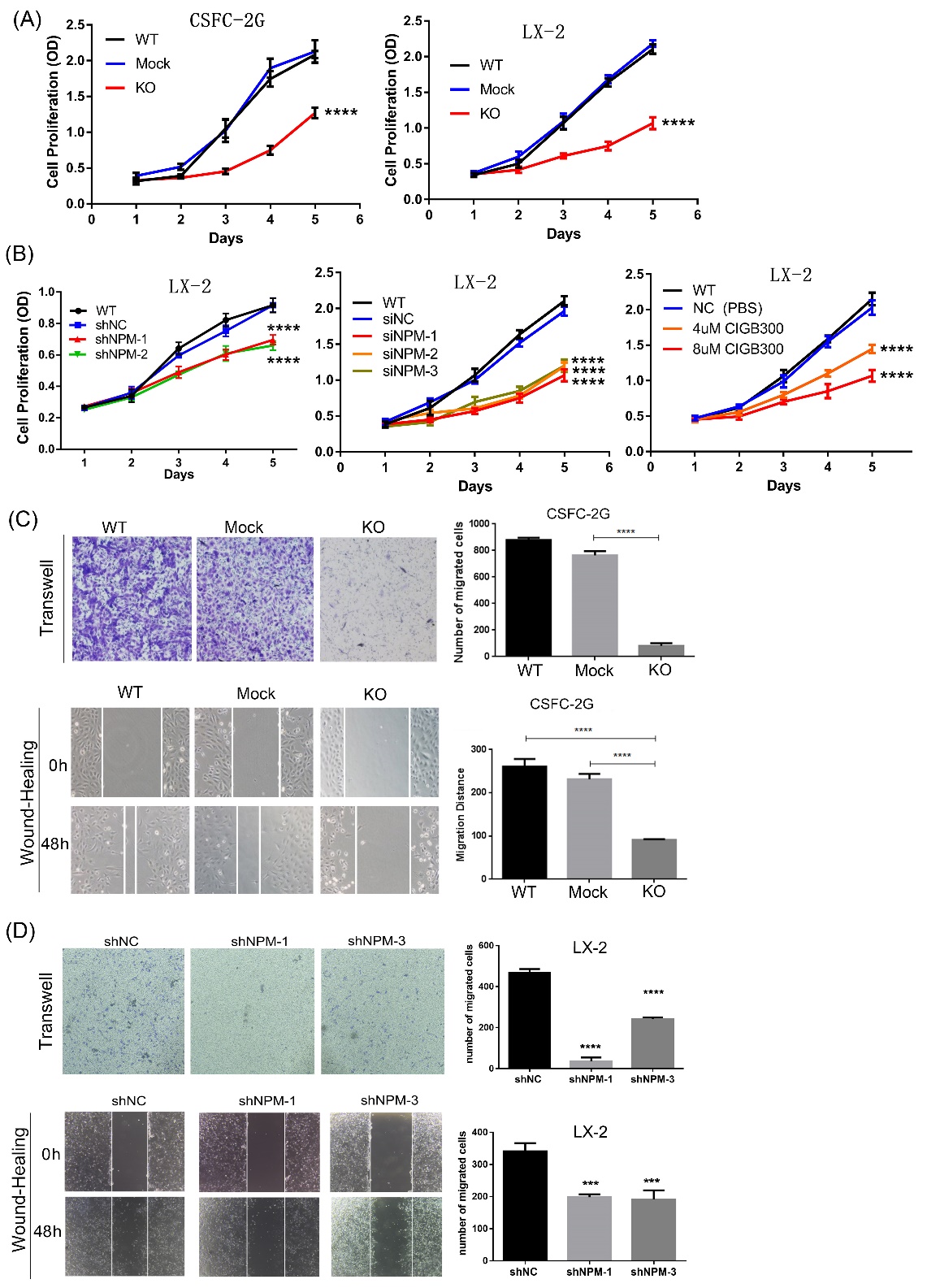


**SFig 1. NPM silencing significantly inhibited the proliferation and migration of hepatic stellate cells.**  (A) CCK-8 proliferation assay showed that NPM knockout inhibited the proliferation of CSFC-2G and LX-2 cells (*n*=3). (B) CCK-8 results indicated that NPM knockdown or CIGB treatment remarkably reduced the proliferation of LX-2 cells (*n*=3). (C) Transwell and Scratch assays showed that NPM knockout inhibited the migration of CSFC-2G cells. (D) Transwell and Scratch assays confirmed that NPM knockdown inhibited the migration of LX-2 cells. Data are expressed as representatives of three independent experiments. *n*=3; mean±SEM; ***p<0.001, ****p<0.0001; T test.

**
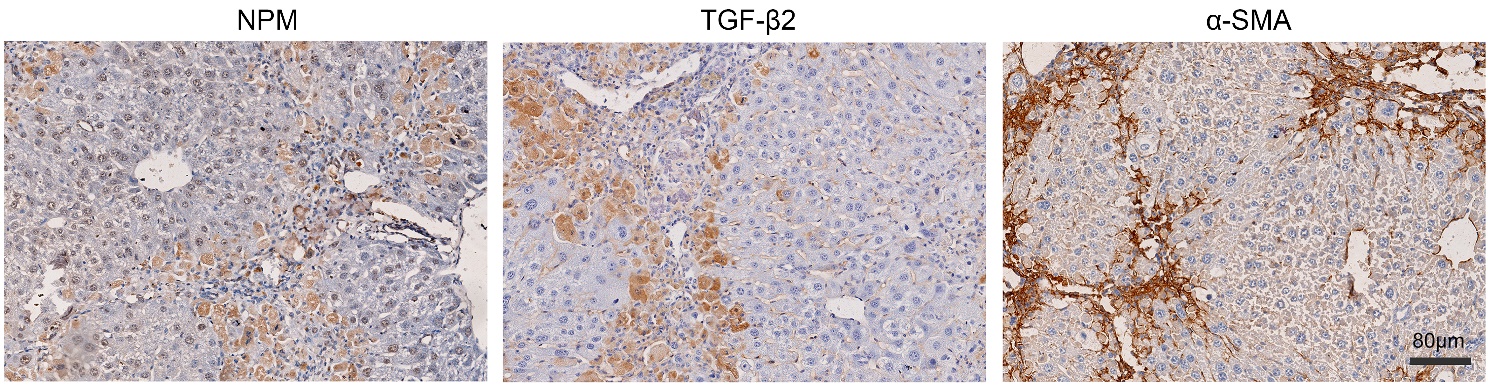
**

**SFig 2. NPM, TGF-β2, and α-SMA have similar distribution in hepatic portal area**. Immunohistochemical staining showed that NPM was expressed in the nucleoli of liver parenchymal cells and was strongly positive in the cytoplasm of injured hepatocytes adjacent to the portal region in the liver tissue of mice with fibrosis. The distribution pattern of hepatocytes with cytoplasmic NPM was very similar to that of TGF-β2 positive cells and α-SMA positive HSCs.


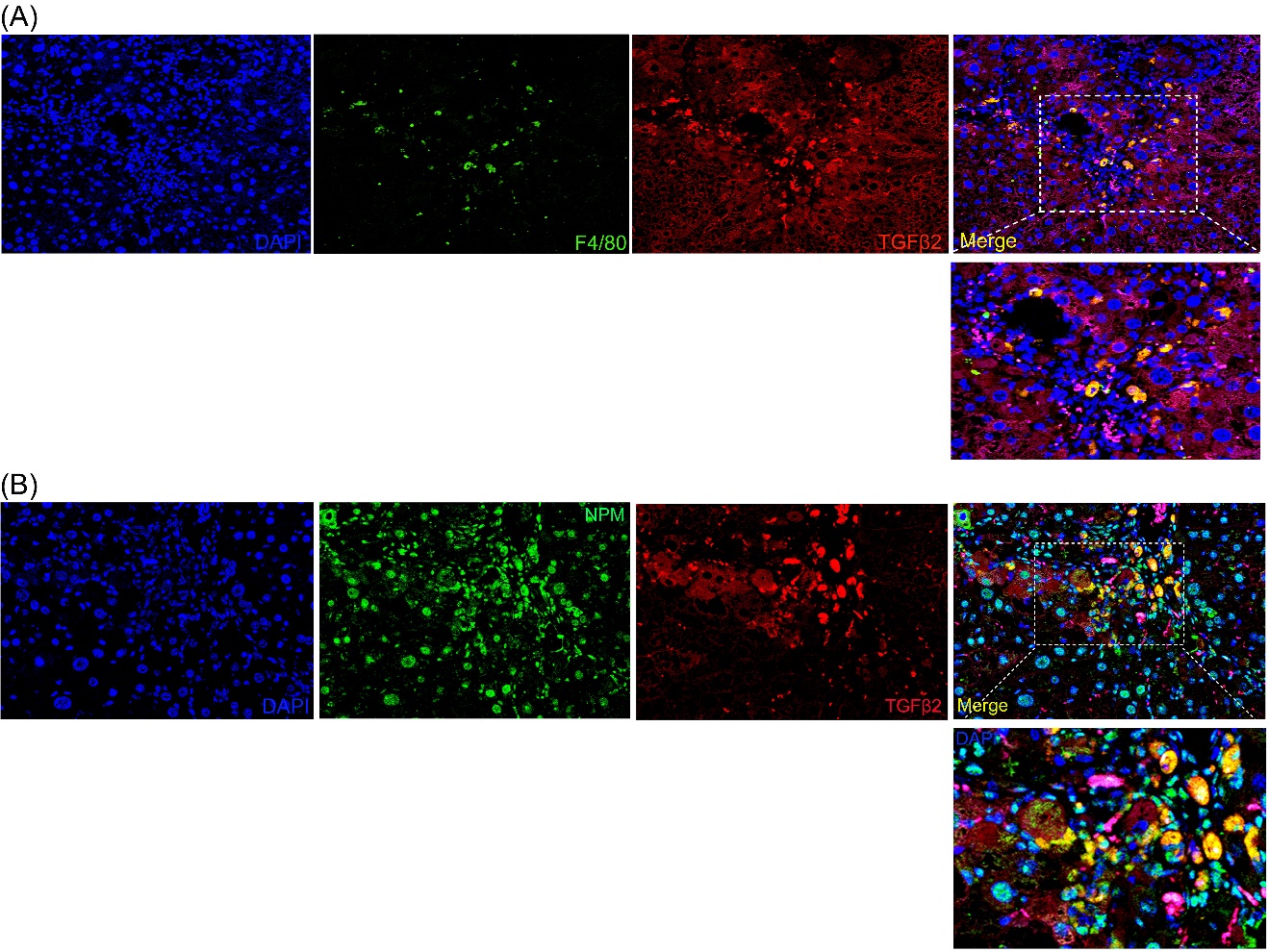


**SFig 3. NPM protein co-located with TGF-β2 protein in Kupffer cells and hepatocytes near the portal region.** (A) Confocal analysis revealed that TGF-β2 protein was strongly expressed in macrophages in the portal region of liver tissue and cells. F4/80 indicates Kupffer cells. (B) Confocal microscopy revealed the co-expression of NPM protein (green) and TGF-β2 protein (red) in the liver portal tract. Yellow indicates the co-localization.

**
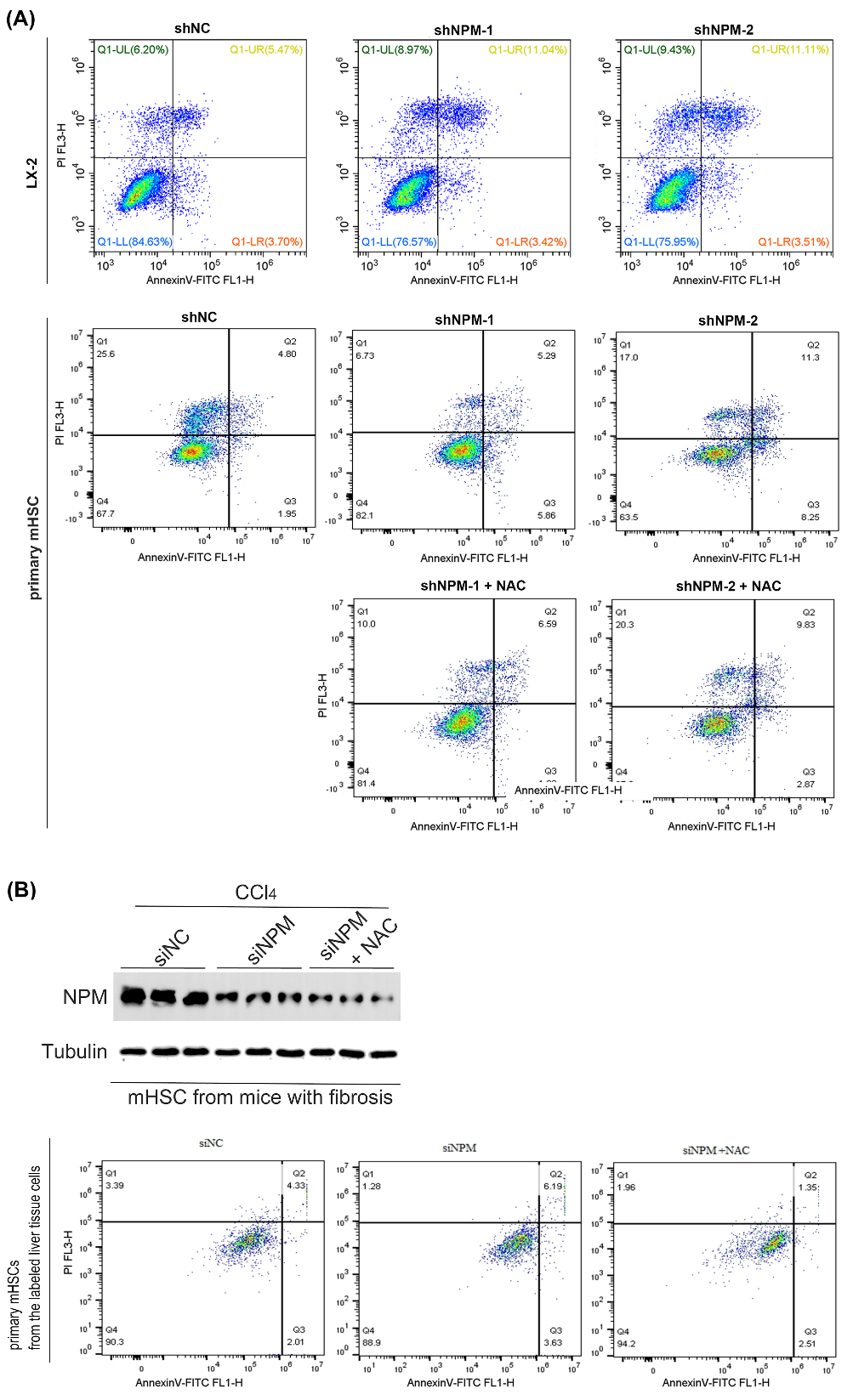
**

**SFig 4. Flow cytometry results showed that NPM inhibition increased apoptosis.** (A) NPM knockdown in LX-2 cells and primary mouse HSCs increased apoptosis. (B) NPM knockdown in mouse hepatic fibrosis models increased apoptosis of HSCs in the liver tissues, and the apoptosis of HSCs was decreased after the upregulated ROS was down-regulated by NAC (n=3 /group).


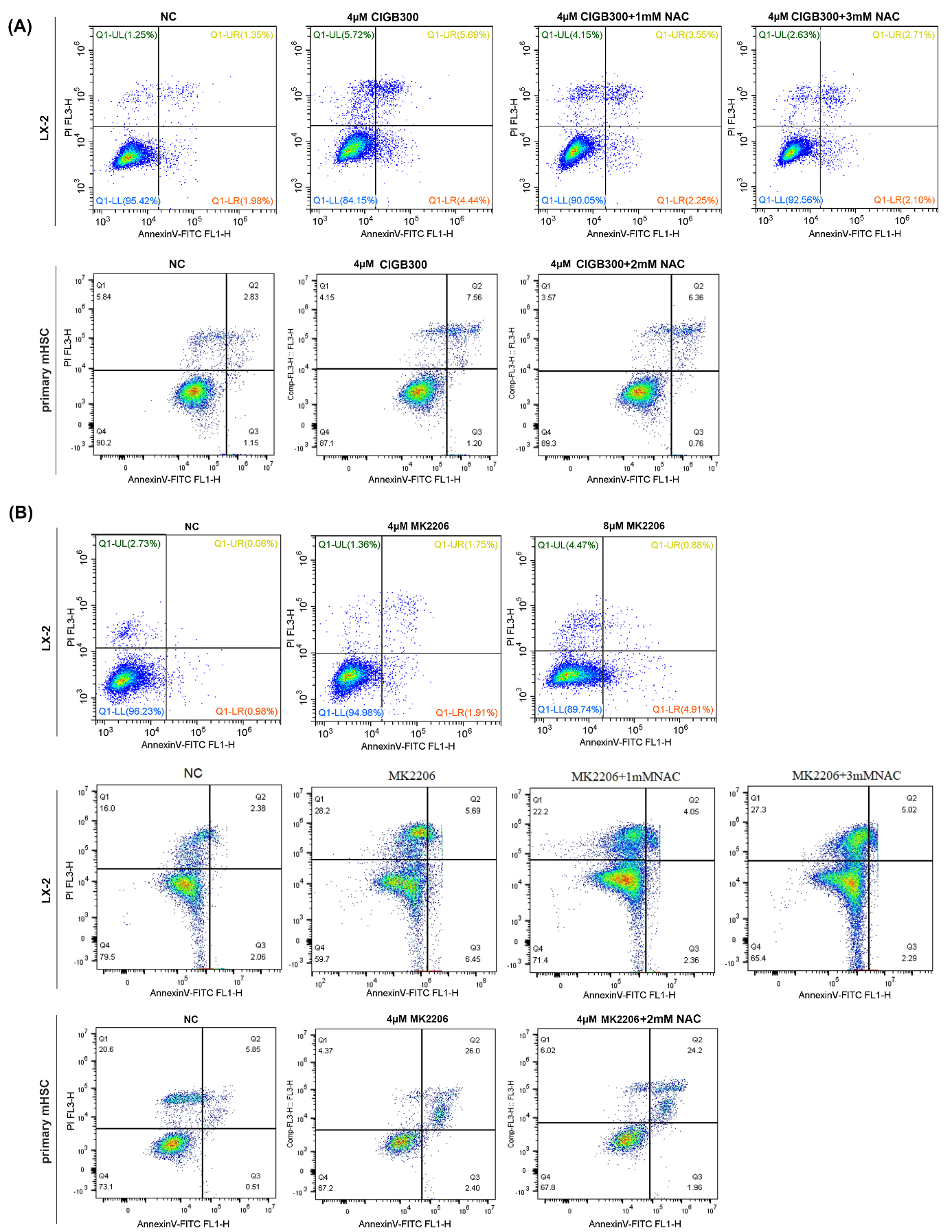


**SFig 5. Flow cytometry results showed that ROS inhibitor N-Acetyl-L-cysteine (NAC) reduced apoptosis in HSC**s**.** (A) NAC reduced CIGB300-induced apoptosis in LX-2 cells and primary mouse HSCs. (B) NAC reduced MK2206-induced apoptosis in LX-2 cells and primary mouse HSCs.

**
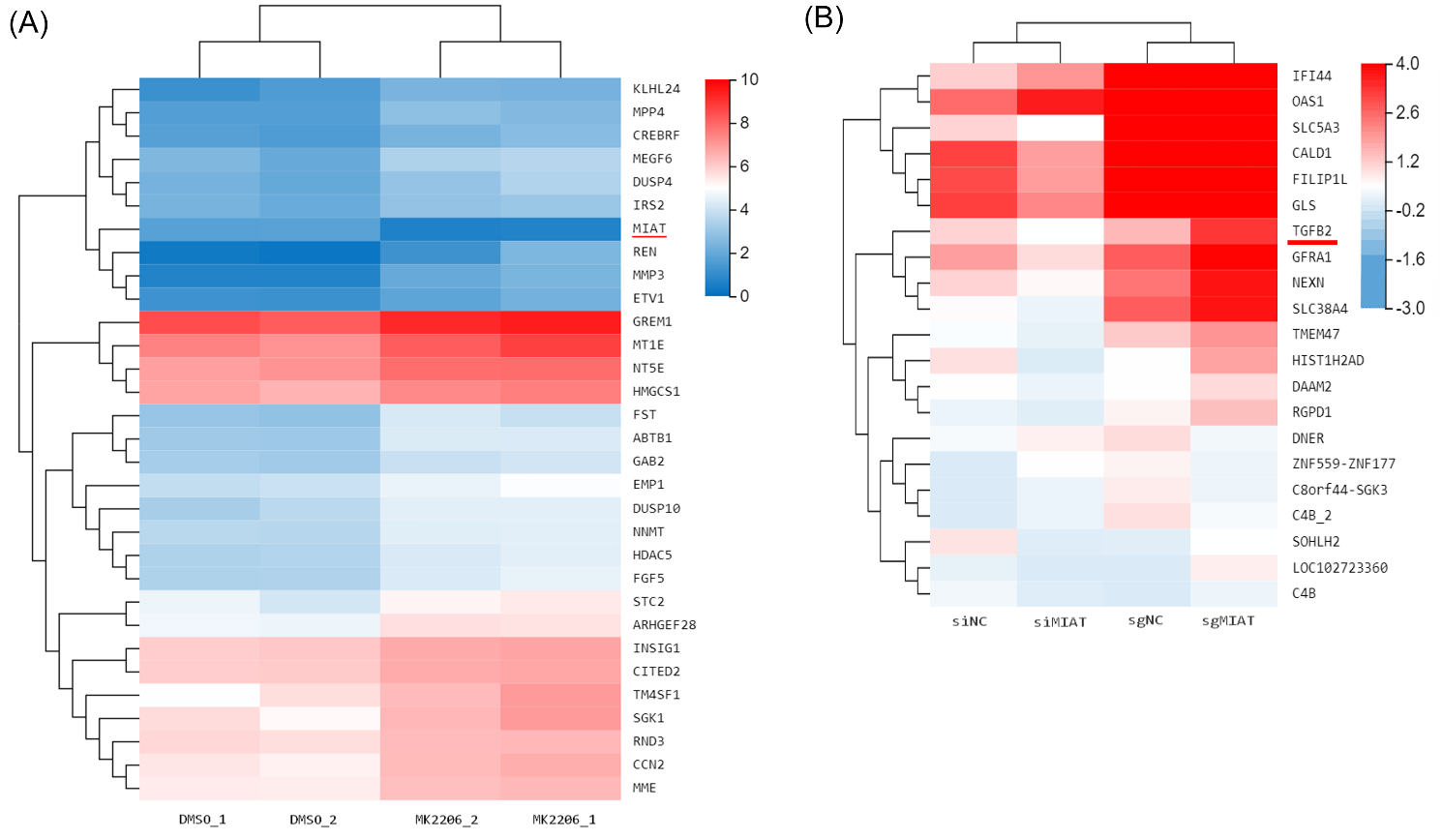
**

**SFig 6. The heatmap of the differentially expressed genes in LX-2 cells regulated by AKT signaling or** **MIAT.**

(A) RNA-seq analysis showed that the AKT inhibitor MK2206 regulated 31 differentially expressed genes in LX-2 cells, including down-regulated MIAT. (B) RNA-seq analysis showed that 21 genes, including TGFB2, were up-regulated or down-regulated by knocking down or activating the endogenous expression of MIAT in LX-2 cells.

**
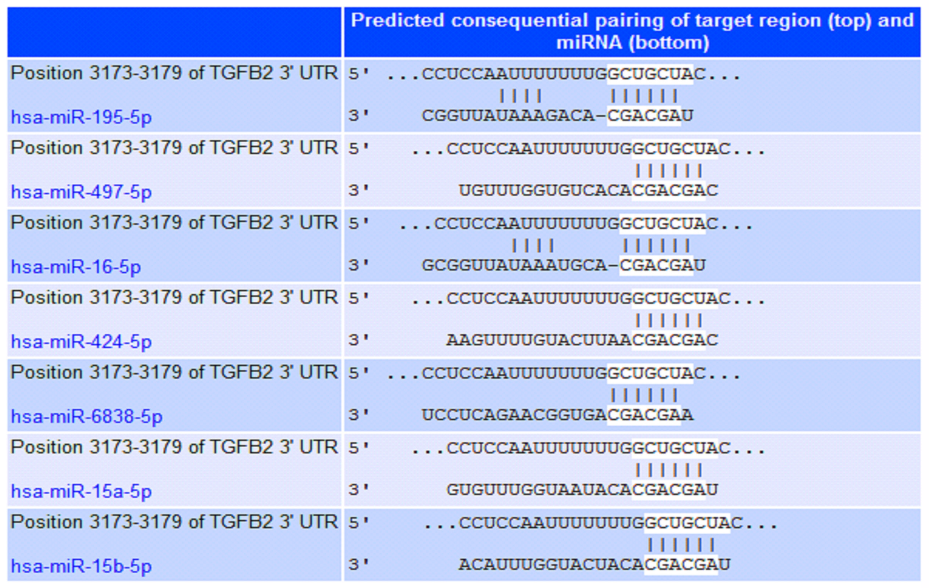
**

**SFig 7. Prediction of binding sites of miRNA on TGF-β2 mRNA.**


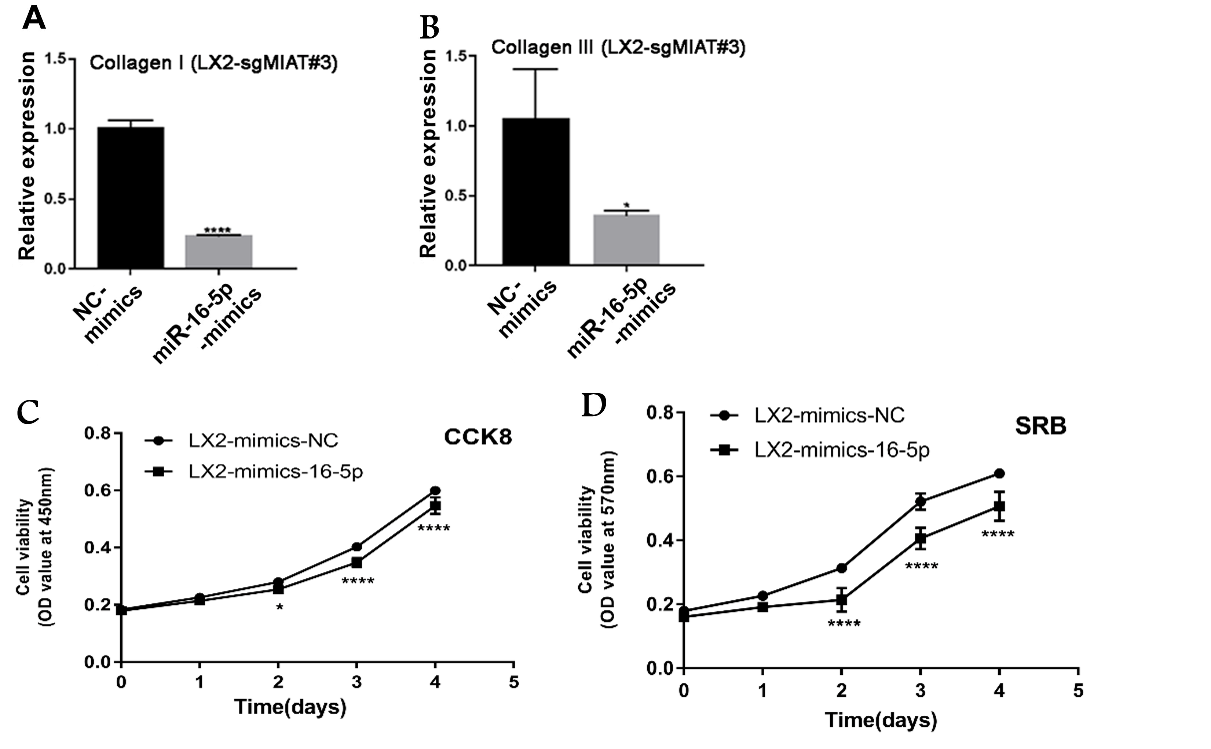


**SFig 8. miR-16-5p mimics downregulated the expression levels of type I and III collagen in LX-2 cells and the proliferation ability of LX-2 cells.**


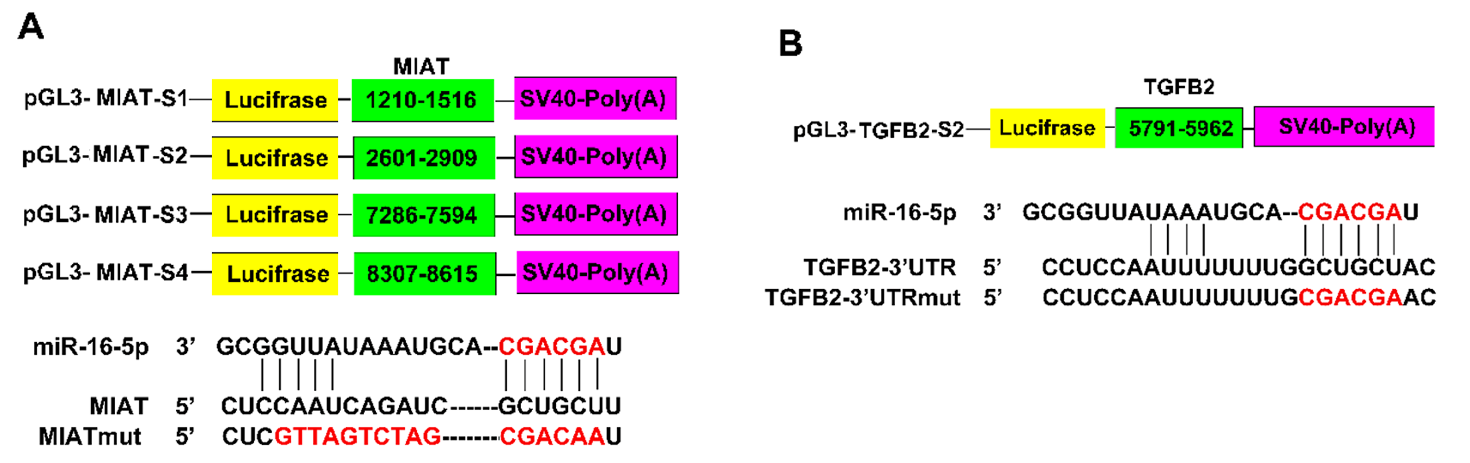


**SFig 9. pGL3 plasmid and miR-16-5p binding sites on MIAT and TGF-β2 mRNA 3' UTR.**

(A) The four nucleotide fragments (approximately 300 bp) predicted to be the binding sites of miR-16-5p on MIAT were inserted into the 3' UTR region behind the coding region of luciferase gene of pGL3 plasmid, and plasmids with corresponding binding site mutations were constructed. (B) The binding site of miR-16-5p was found in the 3' UTR region of the TGF-β2 gene, and the nucleotide fragment of the binding site region (approximately 171 bp) was inserted into the pGL3 plasmid, and the binding site mutation plasmid was constructed.


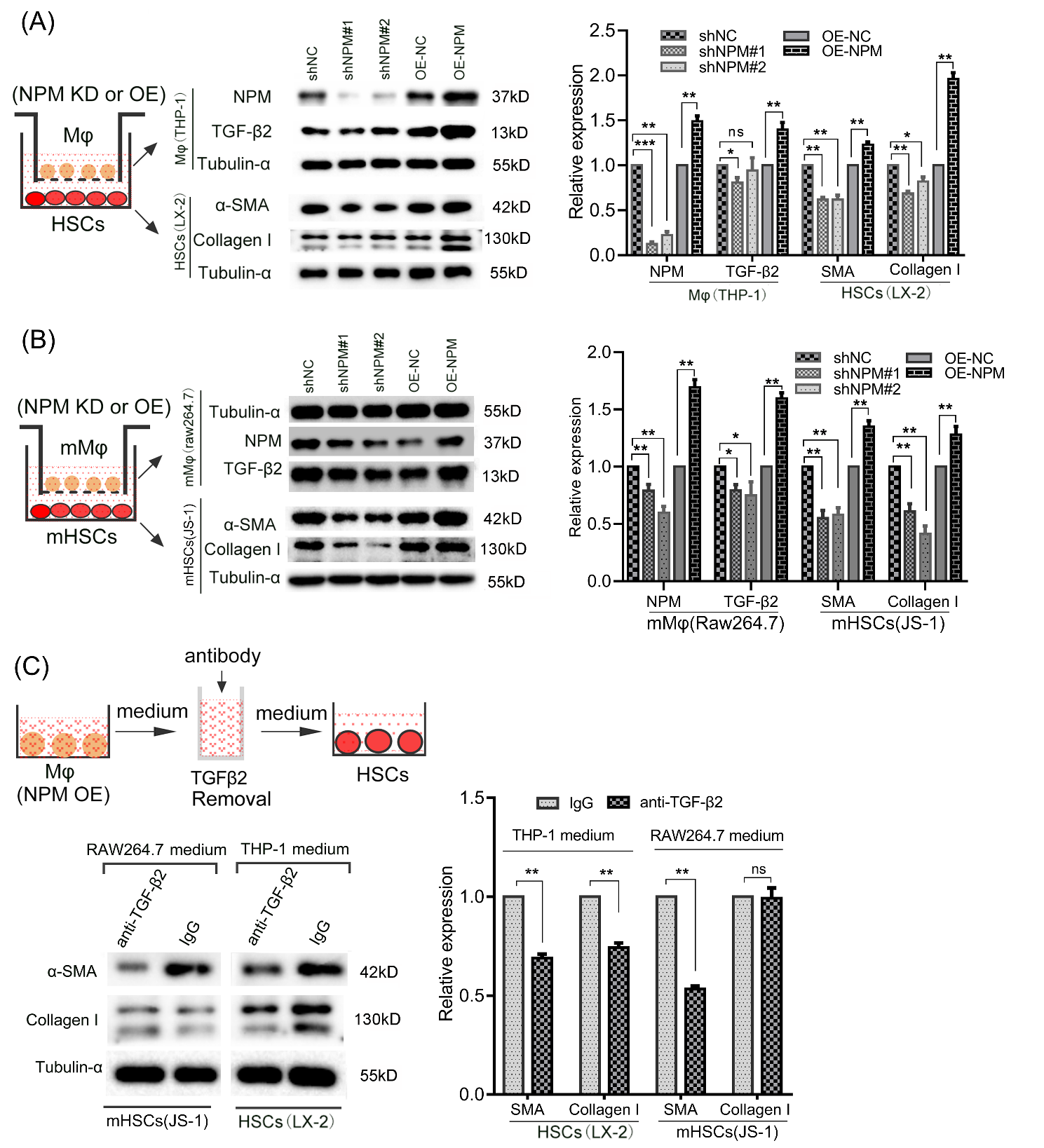


**SFig 10. NPM promotes the secretion of TGF-β2 in macrophages, and the TGF-β2 secreted by macrophages promotes the expression of hepatic fibrosis markers in hepatic stellate cells (HSCs).** (A) Human macrophage THP-1 was co-cultured with human HSC LX-2; (B) Mouse macrophage RAW265.7 was cultured with mouse HSC; and (C) the TGF-β2 protein in macrophage medium supernatant was removed via immunoprecipitation by using the TGF-β2 antibody. This conditioned medium remarkably affected the activation and collagen expression of HSCs LX-2 and JS-1.


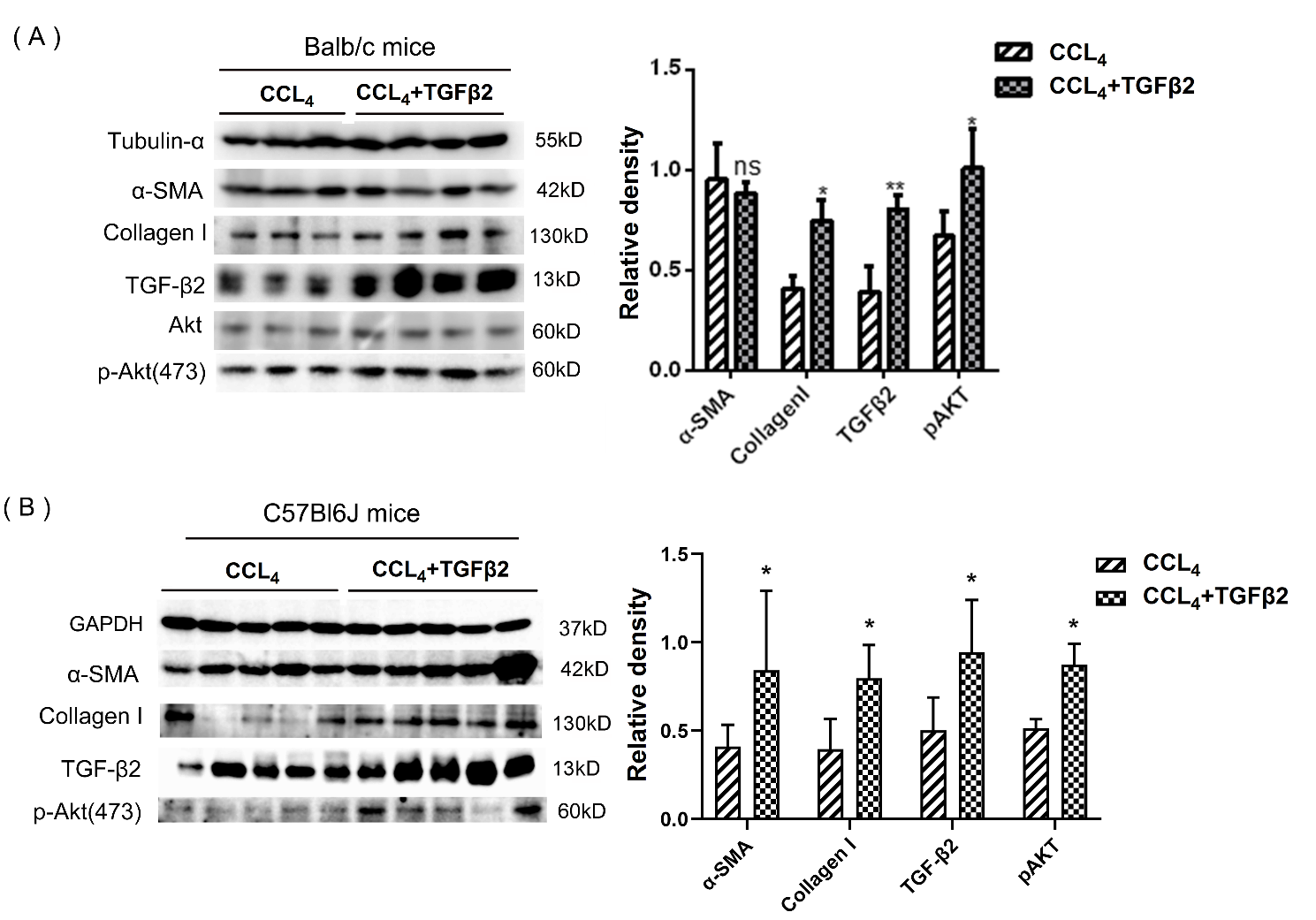


**SFig 11. TGF-β2 increased the expression of liver fibrosis markers in CCl_4_-induced Balb/c mice and C57Bl6J mice** (A) Western blot analysis results confirmed the increased expression of collagen I, NPM, and phosphorylated Akt in the liver tissues of CCl_4_-induced hepatic fibrosis Balb/c mice treated with TGF-β2. (B) Western blot analysis results confirmed TGF-β2 increased liver fibrosis markers collagen I and α-SMA in CCl_4_-induced C57Bl6J mice.

**Table S1. The sequences used for NPM silence.**

| siRNA | Sense (5′ to 3′) | Antisense (5′ to 3′) | |
| --- | --- | --- | --- |
| siNC | UUCUCCGAACGUGUCACGUTT | ACGUGACACGUUCGGAGAATT | |
| siNPM#1 (in vitro) | UGAACUAAAGGCCGACAAATT | UUUGUCGGCCUUUAGUUCATT | |
| siNPM#2 (in vitro) | GUAGCAAGGUUCCACAGAATT | UUCUGUGGAACCUUGCUACTT | |
| siNPM#3 (in vitro) | GUGGAAGCCAAAUUCAUCATT | UGAUGAAUUUGGCUUCCACTT | |
| siNPM-1 (in vivo) | AGAAGCAAUGAACUAUGAATT | UUCAUAGUUCAUUGCUUCUTT | |
| siNPM-2 (in vivo) | AGAGUCUGAAGAUGAAGAUTT | AUCUUCAUCUUCAGACUCUTT | |
| shRNA | Targeting sequences | |  |
| shNC | AATTCTCCGAACGTGTCACGT | |  |
| shNPM#1 | ATGGAATGTTATGATAGGACA | |  |
| shNPM#2 | CCTAGTTCTGTAGAAGACATT | |  |
| shNPM#3 | GCGCCAGTGAAGAAATCTATA | |  |

**Table S2. The sequences used for MIAT activation and silence.**

| sgRNA-MIAT | Targeting sequences | |
| --- | --- | --- |
| sgNC | CTGAAAAAGGAAGGAGTTGA | |
| sgMIAT#1 | TTAATGCGGGAGCGGCTGGC | |
| sgMIAT#2 | GGGGTGCAACCGGTCTTGGA | |
| sgMIAT#3 | AACGCTTGCCCGTCCACATT | |
| sgMIAT#4 | TACTCCGAAGGCTGAGGCGG | |
| shRNA/siRNA | Targeting sequences |  |
| shNC/siNC | AATTCTCCGAACGTGTCACGT |  |
| shMIAT#1/siMIAT#1 | GCTGTGCTCTGACTTACTATT |  |
| shMIAT#2/siMIAT#2 | GGAGTCTACTGAACATCAATT |  |
| shMIAT#3/siMIAT#3 | GGTGTTAAGACTTGGTTTCTT |  |
| shMIAT#4/siMIAT#4 | GCTTTAGATCAGAGTATTCTT |  |

**Table S3. Primary antibodies for WB, IHC and IF.**

| Protein | Concentration  for WB | Concentration  for IHC/IF | Specificity | Company, Cat No. and RRID |
| --- | --- | --- | --- | --- |
| NPM | 1:1000 | 1:300 | Rabbit | Abcam, ab52644; RRID:AB_881735 |
| p-NPM(S125) | 1: 10000 | 1:100 | Rabbit | Abcam, ab109546; RRID:AB_10861473 |
| α-SMA | 1: 2000 | 1:200 | Rabbit | Proteintech, 14395-1-AP; RRID:AB_2223009 |
| β-actin | 1: 20000 | / | Mouse | Proteintech, 66009-1-Ig; RRID:AB_2687938 |
| α-tubulin | 1: 20000 | / | Mouse | Proteintech, 66031-1-Ig; RRID:AB_11042766 |
| Collagen I | 1: 1000 | / | Rabbit | Abcam, ab34710; RRID:AB_731684 |
| MMP-9 | 1: 1000 | / | Rabbit | Abcam, ab38898; RRID:AB_776512 |
| P-Akt | 1: 2000 | / | Rabbit | Cell Signaling Technology, 4060; RRID:AB_2315049 |
| Akt | 1: 1000 | / | Rabbit | Cell Signaling Technology, 4685; RRID:AB_2225340 |
| TGF-β2 | 1: 200 | 1:100 | Mouse | Santa Cruz Biotechnology, sc-374659; RRID:AB_10988781 |
| MMP-2 | 1: 1000 | / | Rabbit | Proteintech, 10373-2-AP; RRID:AB_2250823 |
| Cyclin D1 | 1: 2000 | / | Rabbit | Proteintech, 26939-1-AP; RRID：AB_2880691 |
| GAPDH | 1: 5000 |  | Mouse | Proteintech, 60004-1-Ig; RRID：AB_2107436 |
| F4/80 | / | 1:50 | Mouse | Thermo Fisher Scientific, 14-4801-81; RRID:AB_467557 |
