# Supplementary material for: NPM promotes hepatotoxin-induced fibrosis by inhibiting ROS-induced apoptosis of hepatic stellate cells and upregulating lncMIAT-induced TGF-β2
Source: Cell Death Dis. 2023 Aug 30;14(8):575. doi: 10.1038/s41419-023-06043-0 (PMC10469196; doi:10.1038/s41419-023-06043-0)
Supplement: Supplementary file 2 — Original data files [file 41419_2023_6043_MOESM2_ESM.docx]

**Supplementary material**

Original data files

**
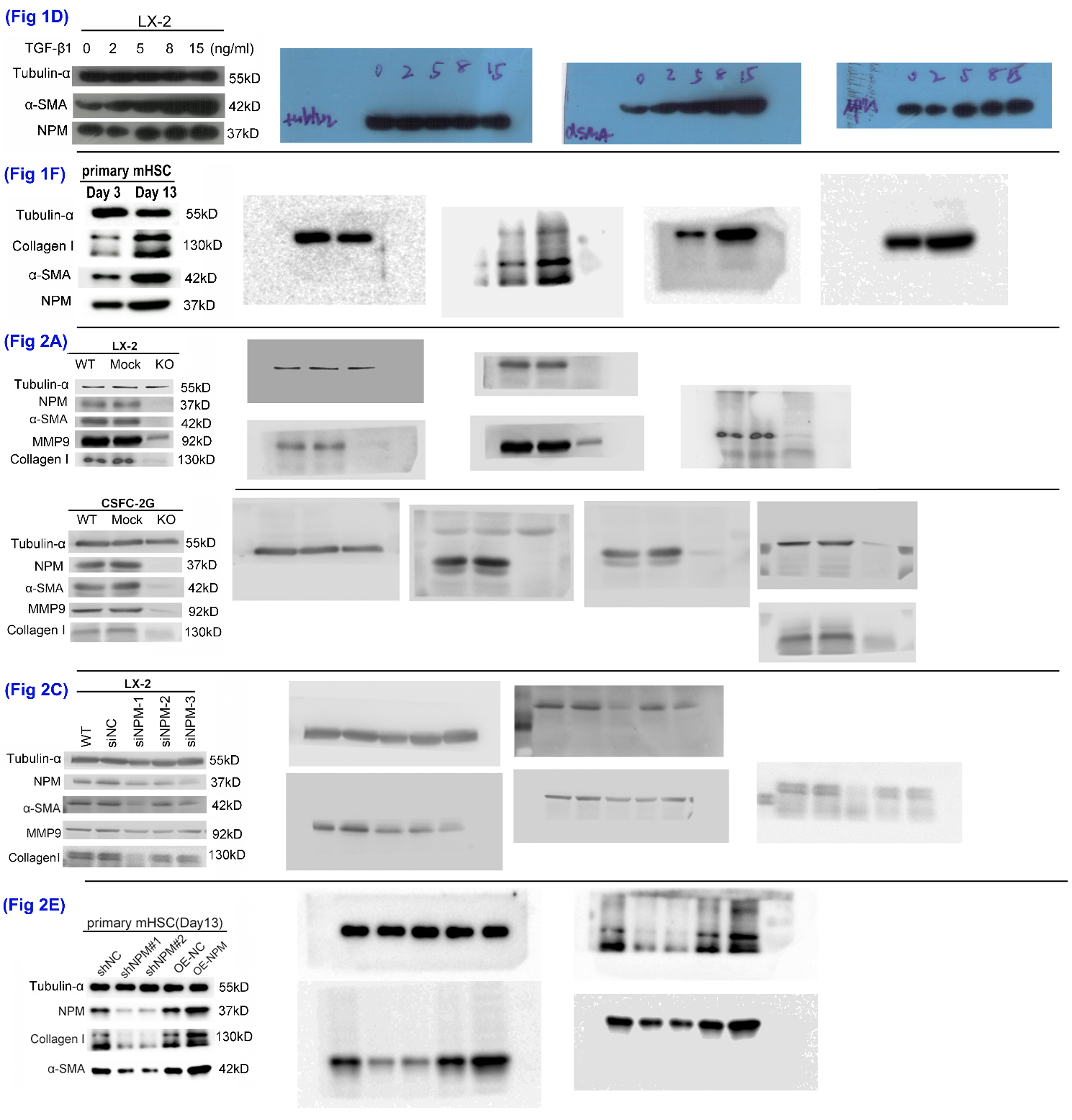
**

**SFig 12-A. The original images corresponding to all WB images in this article (from Fig 1D to Fig 2E).**

**
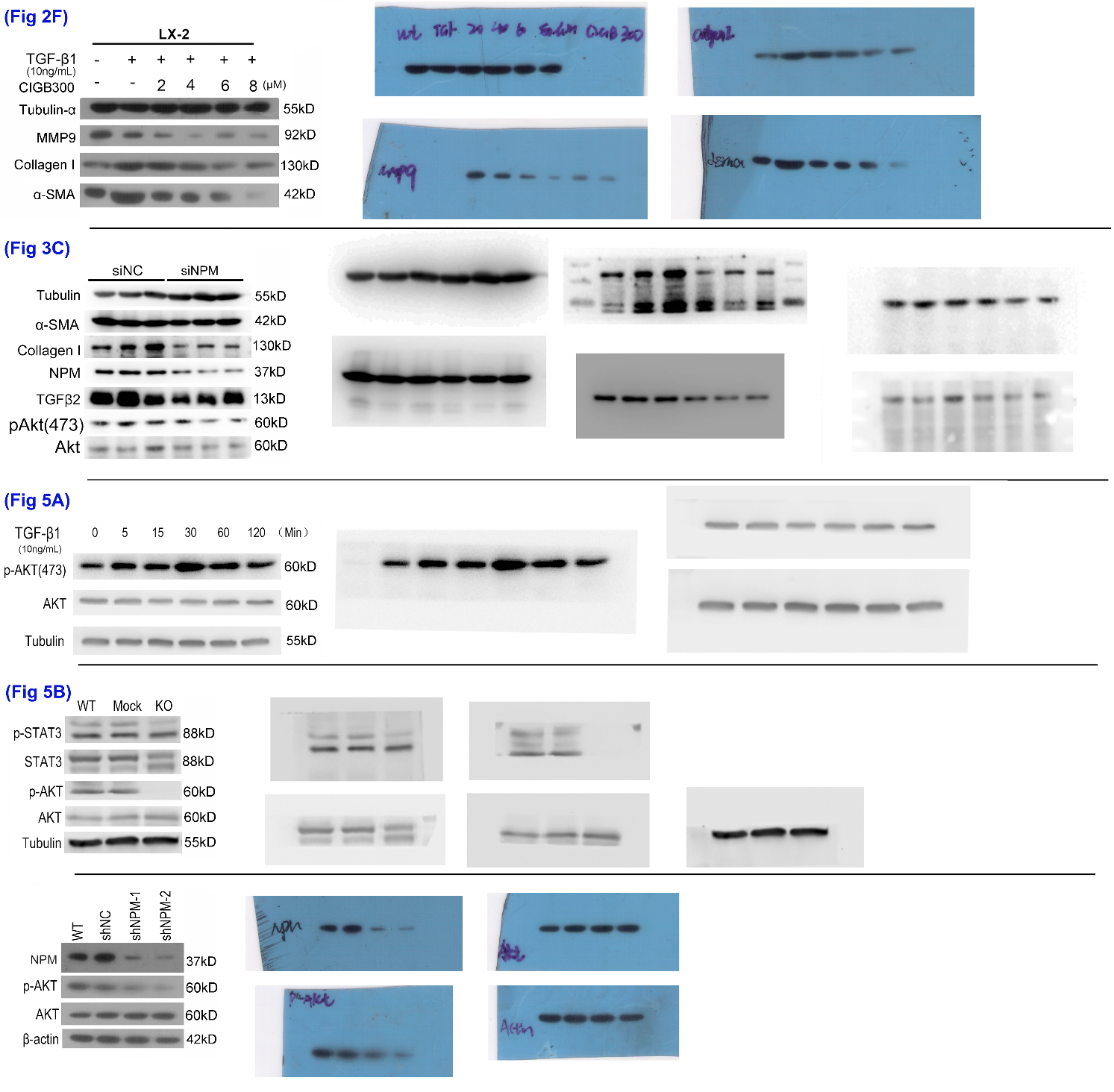
**

**SFig 12-B. The original images corresponding to all WB images in this article (from Fig 2F to Fig 5B).**

**
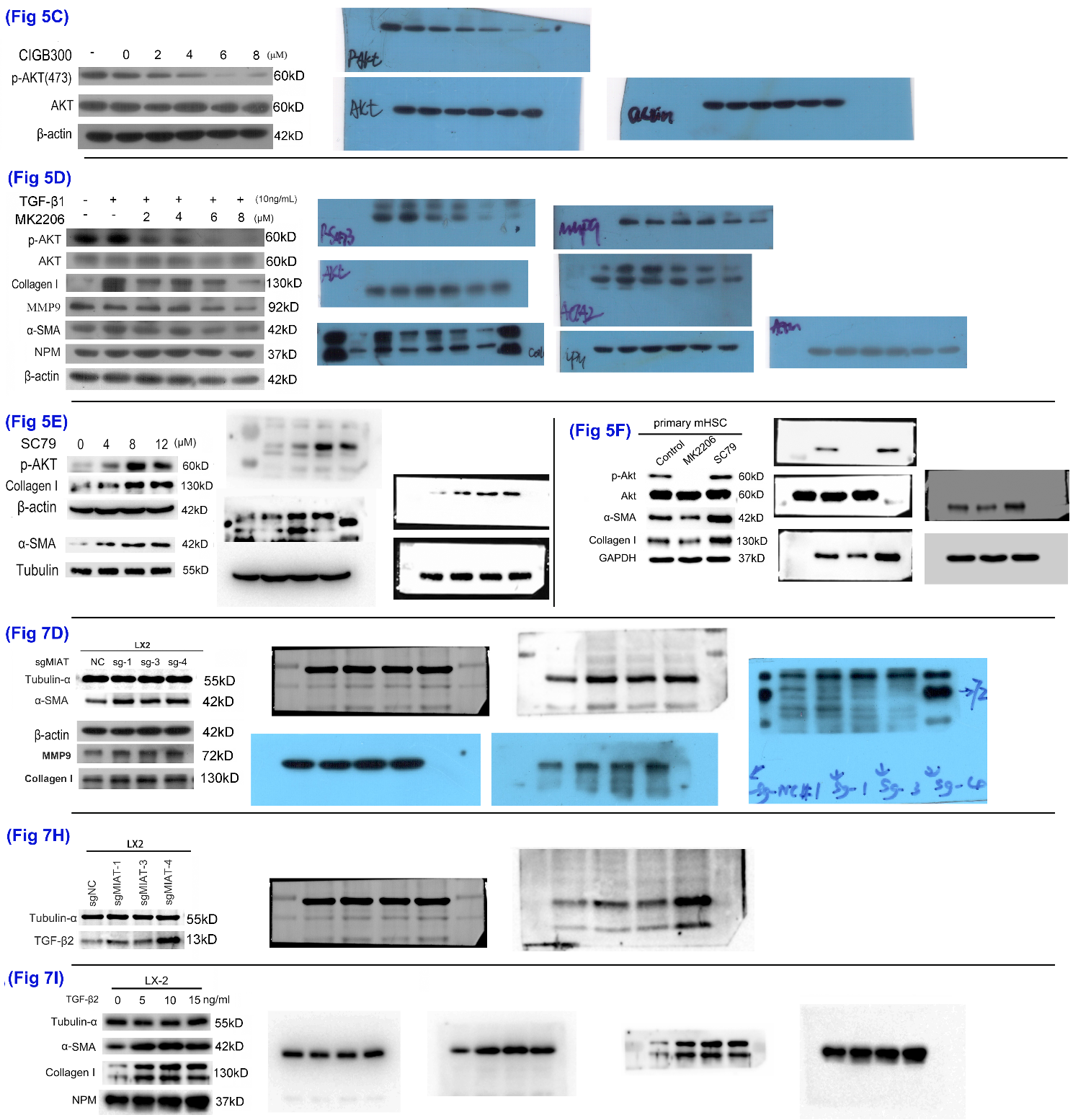
**

**SFig 12-C. The original images corresponding to all WB images in this article (from Fig 5C to Fig 7I).**

**
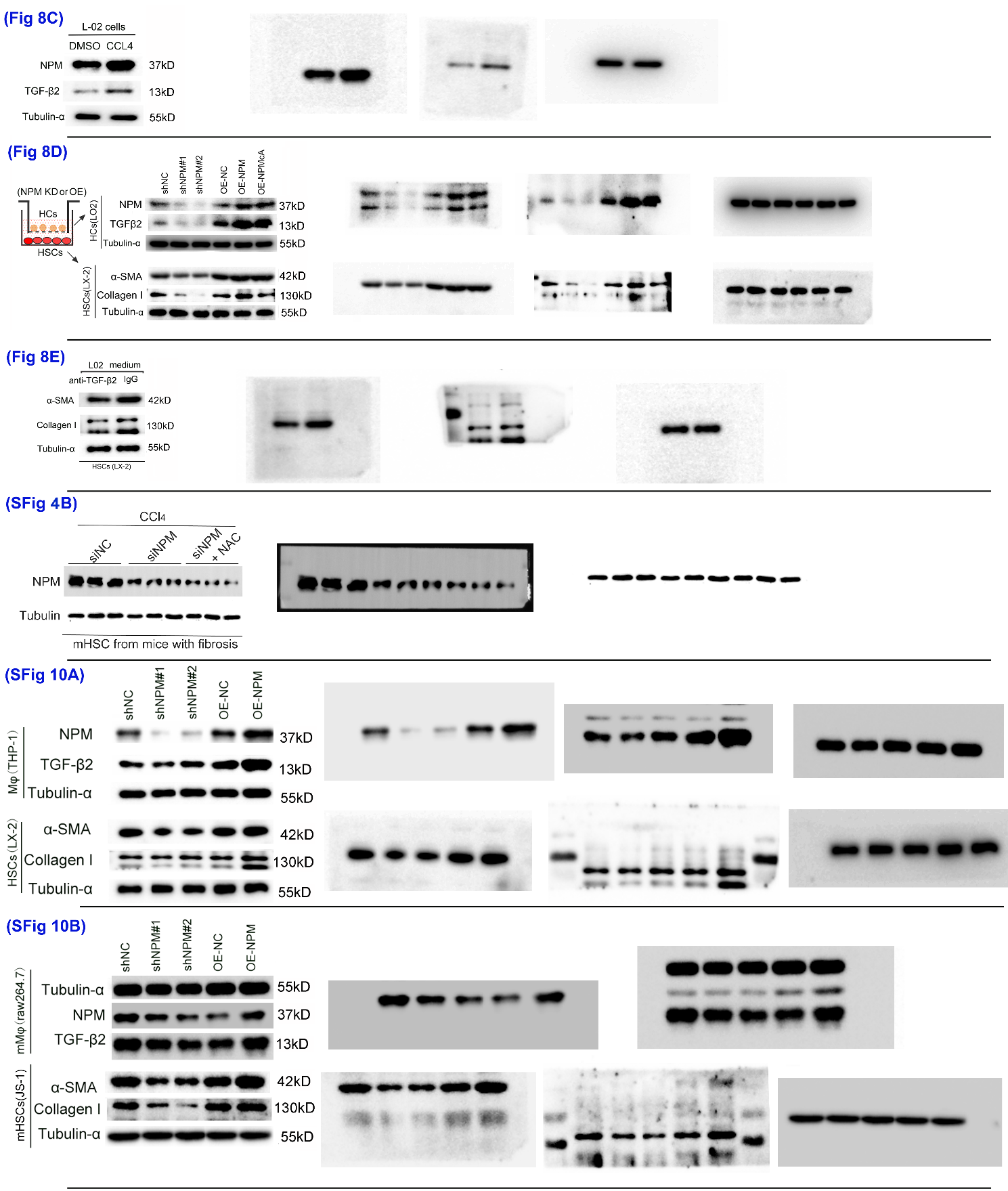
**

**SFig 12-D. The original images corresponding to all WB images in this article (from Fig 8C to SFig 10B).**

**
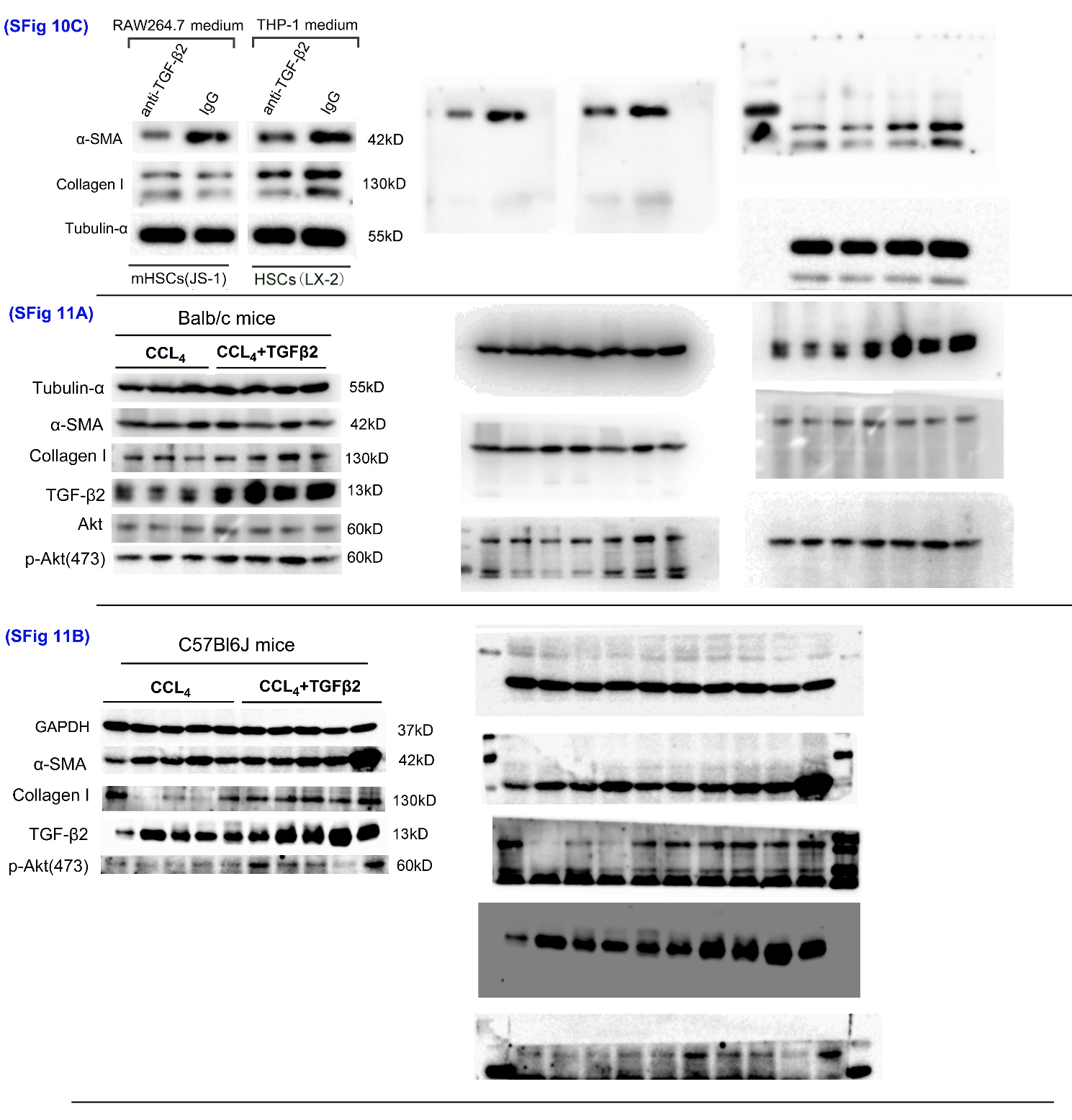
**

**SFig 12-E. The original images corresponding to all WB images in this article (from SFig 10C to SFig 11B).**
